# Implications of SARS-CoV-2 Mutations for Genomic RNA Structure and Host microRNA Targeting

**DOI:** 10.3390/ijms21134807

**Published:** 2020-07-07

**Authors:** Ali Hosseini Rad SM, Alexander D. McLellan

**Affiliations:** Department of Microbiology and Immunology, University of Otago, Dunedin 9010, Otago, New Zealand

**Keywords:** SARS-CoV-2, RNA secondary structure, conserved mutation, miRNA

## Abstract

The SARS-CoV-2 virus is a recently-emerged zoonotic pathogen already well adapted to transmission and replication in humans. Although the mutation rate is limited, recently introduced mutations in SARS-CoV-2 have the potential to alter viral fitness. In addition to amino acid changes, mutations could affect RNA secondary structure critical to viral life cycle, or interfere with sequences targeted by host miRNAs. We have analysed subsets of genomes from SARS-CoV-2 isolates from around the globe and show that several mutations introduce changes in Watson–Crick pairing, with resultant changes in predicted secondary structure. Filtering to targets matching miRNAs expressed in SARS-CoV-2-permissive host cells, we identified ten separate target sequences in the SARS-CoV-2 genome; three of these targets have been lost through conserved mutations. A genomic site targeted by the highly abundant miR-197-5p, overexpressed in patients with cardiovascular disease, is lost by a conserved mutation. Our results are compatible with a model that SARS-CoV-2 replication within the human host is constrained by host miRNA defences. The impact of these and further mutations on secondary structures, miRNA targets or potential splice sites offers a new context in which to view future SARS-CoV-2 evolution, and a potential platform for engineering conditional attenuation to vaccine development, as well as providing a better understanding of viral tropism and pathogenesis.

## 1. Introduction

The SARS-CoV-2 virus has rapidly emerged as a zoonotic pathogen with broad cellular tropism in human or zoonotic-host cells. Host selection pressure on the SARS-CoV-2 virus will have a major impact on the long-term conservation of mutations that enhance viral fitness. Of these selection pressures, the cellular-based adaptive and innate immune systems place constraints on viral fitness. Intracellular detection and anti-viral pathways within infected cells are a critical frontline to control virus replication. The success of the pathogenic SARS coronaviruses is proposed to be due to their ability to suppress intracellular anti-viral pathways [1]. For example, interference with dsRNA detection and the interferon response is enabled through the activity of several non-structural proteins (Nsp). In addition, the sequestration of genomic viral RNA into double membrane vesicles, and dsRNA cleavage by Nsp15, is inferred from the closely related SARS viruses, and likely acts to prevent intracellular detection of the virus [1]. In addition to encoded mechanisms of immune avoidance, the paucity of CpG runs in the SARS-CoV-2 genome with unexpectedly low GC-content at codon position three points to major selection pressure being placed on structural features of the genome [2].

As a recently-emerged zoonotic pathogen, it might be expected that bat-adaptations will not be optimal for infection and replication in human cells. However, extensive mutation and strain-radiation has not yet been observed [3]. The mutation rate in SARS-CoV-2 is reduced by the -proof-reading 3′–5′ exonuclease Nsp14 in the RNA-dependent RNA polymerase (RdRp) complex. The observed mutation rate may be lower than the actual mutation rate, since deleterious mutations have likely been lost through natural selection. The short time frame of SARS-CoV-2 evolution, coupled to a low mutation rate is consistent with a founder effect for geographical bias in mutation patterns [3,4].

A common primary focus of mutational analysis of emerging viruses is the alteration in amino acid sequence of viral proteins that may provide enhanced or new functions for virus replication, immune avoidance, or spread. For instance, the non-synonymous A23403G mutation in the S gene may enhance viral infectivity through decreased S1 shedding and increased S trimer stability [5]. However, synonymous mutations can critically impact nucleic acid secondary structure and sub-translational events including genome replication and packaging, and virus maturation [6,7], as well as translation and polypeptide folding [8,9]. In addition, the RNA secondary structures of SARS-CoV-2 genes have been proposed to be druggable targets [10,11,12]. Because little is known of the influence of SARS-CoV-2 mutations on the RNA secondary structure, and its possible implications for inhibition by host miRNA, we have modelled the impact of common mutations of the SARS-CoV-2 RNA structure and the susceptibility of the genome to interference from host miRNA.

The incident presence of host miRNA targets within the SARS-CoV-2 genome may be pivotal for host selection pressures to further shape further viral evolution. Viruses not only alter host miRNA expression, but may also produce miRNAs to promote their infectivity [13,14,15,16]. On the other hand, the host targets viral transcripts for inhibition of translation, or mRNA destruction, through a miRNA-mediated defence system. Since miRNAs are divergent between species [17], it would be expected that bat-adapted SARS-CoV-2 will undergo selection pressure derived from human miRNA interference [13,14,15,18,19]. While perfect matches of miRNA to target viral sequences result in miRNA-induced silencing complex (miRISC)-mediate destruction of viral RNA, imperfect matches interfere with translation [20].

A growing body of evidence suggests that human miRNAs act as a critical host defence against coronaviruses. An interaction between human coronavirus OC43 nucleocapsid and miR-9 can enhance the type I interferon response necessary to clear viral infection [21]. Several host miRNAs (miR-574-5p, −214, −17, −98, −223, and −148a) bind to SARS-CoV encoded transcripts such as S, E, M, N, and ORF1a [22,23]. However, SARS-CoV escapes from miRNA-mediated defence through the manipulation of host miRNA machinery [22,23]. Additionally, SARS-CoV and SARS-CoV-2 express short RNAs that resemble miRNAs and could impact upon host house-keeping or immune defence processes [24,25,26]. More recently, several studies have proposed that host miRNAs bind SARS-CoV-2 transcripts [24,26,27]. However, the relevance of host miRNAs for inhibition of viral replication is relevant only if the identified miRNAs are expressed in target host cells.

Both DNA viruses, and ‘cytoplasmically-confined’ RNA viruses, use the host RNA splicing-machinery to generate new viral transcripts, or to modify the host transcriptome in favour of their own replication [28,29,30,31,32]. It has been suggested that the fused leader sequence in 5′ end of the mouse hepatitis virus (betacoronavirus) mRNAs is the result of a non-canonical splicing process [33]. Moreover, deep RNA sequencing has identified several unknown SARS-CoV-2 viral RNAs, possibly the result of non-canonical splicing events [34]. Therefore, our study has additionally identified and mapped mRNA splice sites within the SARS-CoV-2 genome.

No selective advantage of the identified sequence alterations in SARS-CoV-2 should be inferred by their inclusion here. However, the potential of these mutations to impact upon RNA structure and miRNA recognition provides a basis for ongoing monitoring of viral evolution at these sites in the SARS-CoV-2 genome.

The interplay of viral genome sequences and host miRNA is translatable for clinical outcomes. For example, the inclusion of host miRNA binding sites into the ORF of conserved viral regions essential for the viral life cycle is a feasible mechanism for the attenuation of live vaccines [35,36,37,38].

## 2. Results

### 2.1. Identification of SARS-Cov-2 Recurrence Mutations

A total of 65 SARS-CoV-2 patient isolate sequences were collected from NCBI and GISAID databases and aligned against SARS-CoV-2 reference sequence NC_045512.2 (Table S1). The mutations present in multiple sequences and in at least in three different countries were categorized as ‘conserved mutations’ (Table 1) [39].

**Figure 1:**
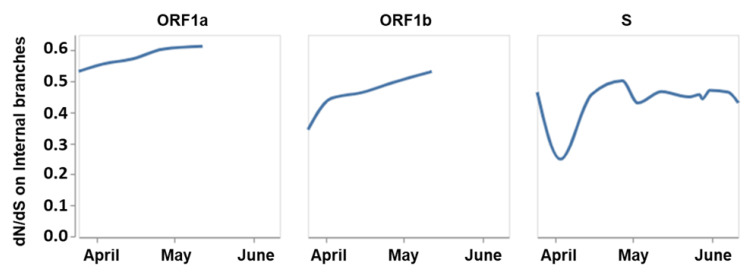
The ratio of SARS-CoV-2 nonsynonymous to synonymous mutations obtained from the Observable notebook (sequencing data available up to June 12, 2020).

Greater than 50% of the observed mutations in our analysis were synonymous mutations (Figure 1, Table S2). Similar data was obtained from Observable notebook on all sequencing data available up to 12 June 2020 (Figure 1, Table S2). Recently, Li et al. suggested that SARS-CoV-2 is under purifying selection, with dN/dS < 1 [40]; similar results were observed in our study and others [40,41].

Most of these mutations are substitutions of C/G to U. The high A/U content (U = 32.1%; A = 29.9%; G = 19.6%; C = 18.4%) and enrichment of codons in pyrimidines is likely due to APOBEC editing of viral RNA and the fact that the proof-reading Nsp14 does not remove U (the product of cytosine deamination) [42]. Two mutations at 241 and 29742, are in the 5′ and 3′ untranslated regions (UTRs). Nine mutations are synonymous mutations, including 313, 3037, 9802, 9803, 14,805, 17,247, and 28,686, while the others are non-synonymous (Table 1). Interestingly, the C27964U (S24L in ORF8) exists only in 97 USA sequences, with the earliest isolated on March 9th (MT325581.1), after USA underwent lockdown [43] (Figure S1).

### 2.2. RNA Secondary Structure

**Figure 2:**
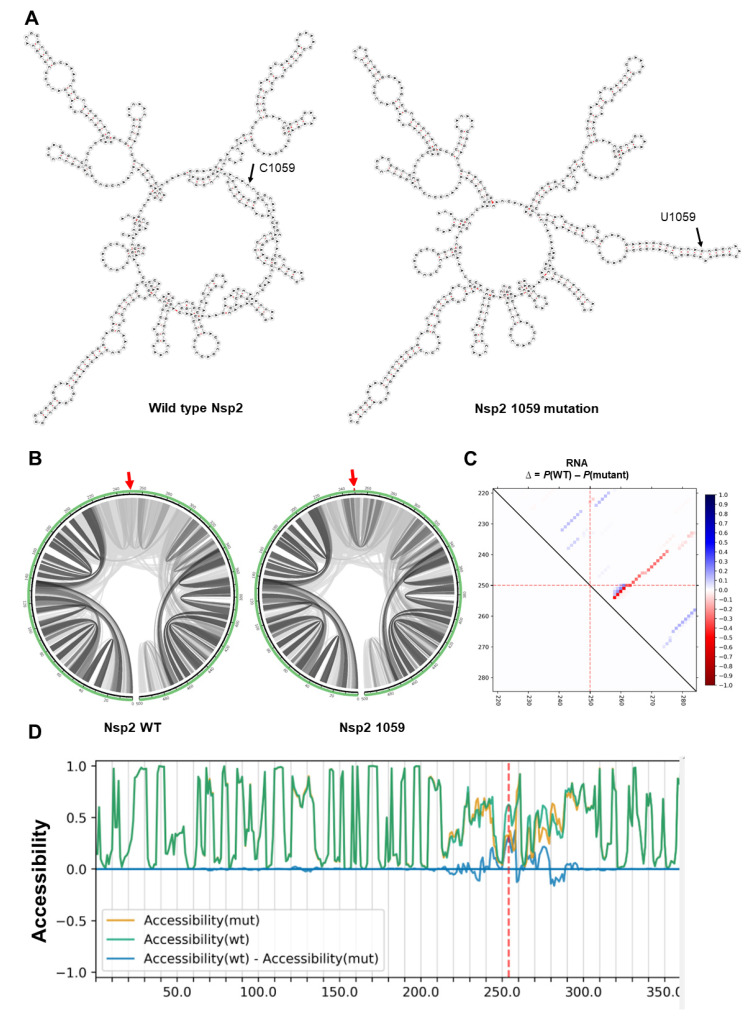
The impact of C1059U mutation on local RNA secondary structure of Nsp2. (**A**) RNA secondary structures of Nsp2 wild type (MFE structure: −146.10 kcal/mol—centroid structure: −132.30 kcal/mol) and 1059 mutation (MFE structure: −147.20 kcal/mol—centroid structure: −137.80 kcal/mol) using RNAfold tool. (**B**) The base pair probabilities by circular plots with higher base pairing potential is reflected in darker hues of grey lines and the mutated position highlighted by red arrow (MutaRNA). (**C**) The dot plot shows the differences of the base pairing probabilities of 1059 mutation vs. wild type RNA, Pr(bp in WT)—Pr(bp in mut). The base pairs weakened by the 1059 mutation are in blue, while higher base pair probability in the mutant is depicted in red. The mutated position is highlighted by red dotted lines (P values based on RNAsnp are as follows: mode-1 = 0.2617, mode-2 = 0.3344). (**D**) The accessibility profiles of wild type (green line) and the mutation (yellow line) and their differences provide an assessment of the mutation effect on the RNA single-strandedness, which may relate to its interaction potential with other RNAs or proteins. Accessibility is measured in terms of local single-position unpaired probabilities and is plotted as WT—Mut, whereby a negative value indicates increased accessibility caused by the mutation [45]. The mutated position is highlighted by a red line.

Among all the mutations, only two mutations were predicted to have an impact on the secondary structure of viral RNAs. First, a conserved mutation 1059 in Nsp2 changed the secondary structure of Nsp2 dramatically (Figure 2A). We performed local RNA secondary structure analysis on 500 bp flanking the mutation region (250 bp upstream and 250 bp downstream of mutation site), as global folding predictions for large mRNA have been shown to be unreliable [44].

Next, the effects of mutations on base pair probabilities of local folding of Nsp2 RNA were investigated. As shown in Figure 2B, the 1059 mutation increased the Watson–Crick base pair probability in flanking regions, resulting in a more stable predicted RNA secondary structure (Figure 2C). The 1059 mutation had no effect on RNA accessibility which is a consideration for RNA-RNA and RNA-protein interactions (Figure 2D).

**Figure 3:**
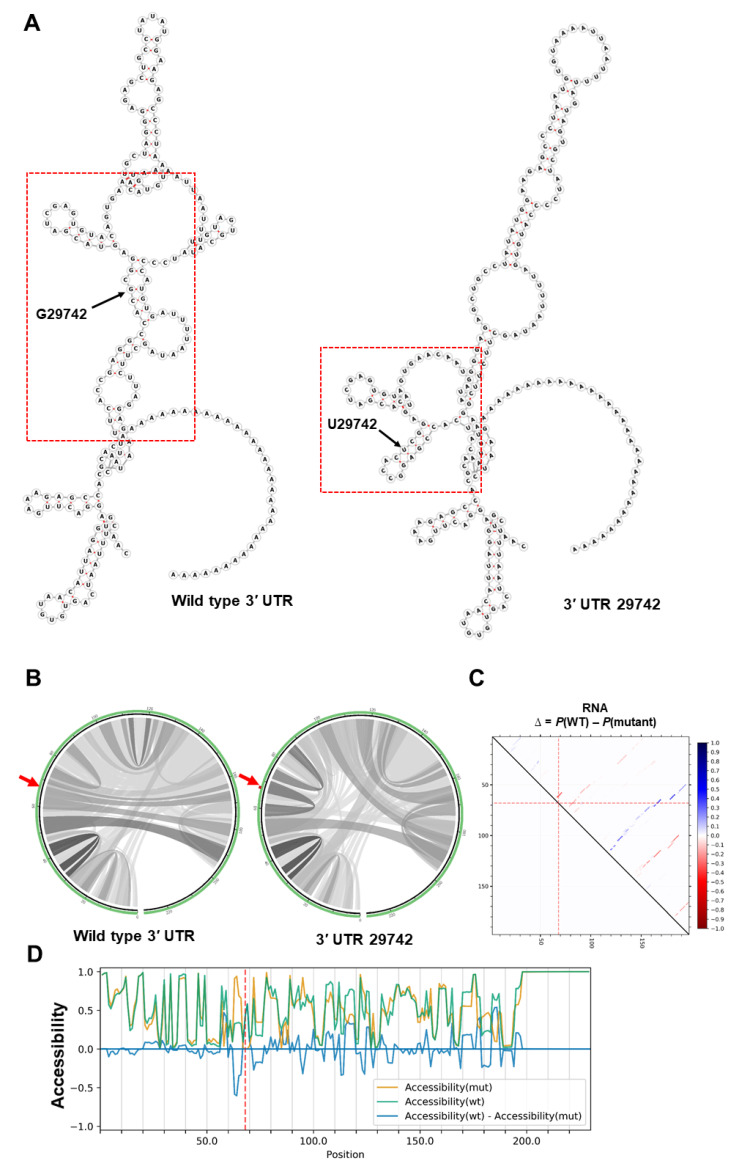
The impact of G29742U mutation on the 3′ UTR. (**A**) The RNA secondary structures of wild type 3′ UTR (MFE structure: −36.90 kcal/mol—centroid structure: −30.50 kcal/mol) and 29,742 mutation (MFE structure: −40.30 kcal/mol—centroid structure: −30.30 kcal/mol) using RNAfold tool. Note the change in predicted secondary structure of 3′ UTR RNA through the 29742 mutation. The s2m regions are highlighted by red rectangles. (**B**) The base pair probabilities of global fold of Nsp2 RNA demonstrated by circular plots, with higher base pairing potential reflected in darker hues of graduated grey lines. The original and mutated nucleotides are highlighted by red arrows (MutaRNA). (**C**) The dot plot shows the differences of the base pairing probabilities of the 29,742 mutation vs. wild type RNA, Pr(bp in WT)—Pr(bp in mut). The base pairs weakened by the mutation are in blue while higher base pair probability in the mutant is depicted in red. The mutated position is highlighted by red dotted lines (P values based on RNAsnp are as follows: mode-1 = 0.6204, mode-2 = 0.6638). (**D**) The accessibility profiles of wild type (green line) and mutation (yellow line) and their differences provide an assessment of the effect of the mutation on the RNA single-strandedness. Accessibility is measured in terms of local single-position unpaired probabilities and is plotted as WT—Mut, whereby a negative value indicates increased accessibility caused by the mutation [45]. The mutated position is highlighted by a red line.

Mutation 29742 occurs in a conserved region within 3′ UTR known as the coronavirus 3′ stem-loop II-like motif (s2m). This mutation alters the global RNA secondary structure of the 3′ UTR (Figure 3A). An increase in stability of s2m in the mutated sequence was observed in both MFE (−6.10 kcal/mol vs. −11.70 kcal/mol) and centroid (−0.47 kcal/mol vs. −11.40 kcal/mol) structures. It is well known that s2m is present in most coronaviruses and plays a vital role in viral replication and invasion [46,47,48]. Mutations in this region have been shown to increase the stability of 3′ UTR and its interaction with 5′ UTR [47].

Analysing base pairing probability, the G29742U mutation slightly decreased base pair probabilities in the global folding of RNA (Figure 3B). But the same mutation slightly increased the number of strong base pair probabilities downstream of the mutation in the s2m region 29795-29865 (Figure 2C) and may contribute to the stronger thermodynamic structure predicted in mutated s2m (see above). Several SARS-CoV-2 encoded genes bind to the host proteins involved in biological processes, such as protein trafficking, translation, transcription, and ubiquitination regulation [49,50]. In addition, s2m interacts with viral and host proteins such as the polypyrimidine tract-binding protein (PTB), to regulate viral replication and transcription [47,48]. Interestingly, the G29742U mutation (underlined) removed a c-Myc binding site (GCC ACG CGG A) within s2m, but increased the RNA accessibility of this region (Figure 3D).

It should be noted that both 1059 and 29,746 mutations exist in the regions that are highly sensitive to nucleotide changes based on the RNAsnp mode-3 and RaSE programs (Tables S3–S6). Collectively, these results suggest that 1059 and 29,742 yield more stable RNA structures around the mutation sites. However, noting the limitations of prediction software, the relationship of changes in RNA secondary structure of Nsp2 and 3′ UTR to viral replication or infectivity must be tested in adequate experimental assays.

### 2.3. Potential Interaction of SARS-CoV-2 Transcripts and Human miRNAs

Using databases and published data, we filtered our considered miRNA to those with documented expression in SARS-CoV-2 target cells, and additionally focused on miRNAs that have been reported as components of the anti-viral miRNA-mediated defence system. Using independent programmes, we identified ten human miRNAs with potential binding sites across the SARS-CoV-2 genome (Figure 4 and Figures S3–S17).

As shown in Figure 5, a total of eight mutations were detected in six miRNA binding sites of which four are conserved mutations (3037, 9802, 9803 and 24034).

Out of eight mutations, two mutations are G↔A, while six mutations are G/C→U, likely the result of host RNA editing mechanisms [51]. We hypothesised that some mutations may affect the miRNA binding sites and therefore impact on miRNA-mediated defence, since miRNA-mRNA interactions are sensitive to the GC loss (see above). We also mapped the critical positions in which nucleotide substitutions will negatively affect miRNA binding to its target (Figure 5, asterisks).

MiR-197-5p is upregulated in patients with cardiovascular disease and has been proposed as a biomarker for the prediction of cardiovascular events [52,53,54]. It is well established that patients with cardiovascular disease are overrepresented in symptomatic COVID-19 cohorts and have a higher mortality rate [55]. The C3037U conserved, but synonymous, mutation within Nsp3 sequence abolished the miR-197-5p target sequence, as the C3037 nucleotide is among the sensitive nucleotides (Figure 5, Table S7). This mutation was introduced in early January 2020 (Figure S2), and is frequently linked to dominant D614G mutation [56]. Interestingly an analysis carried out by van Dorp et al. showed that C3037U mutation is a homoplasy that has independently emerged 39 times in global lineages and has a positive association with clade expansion [4]. 

**Figure 4:**
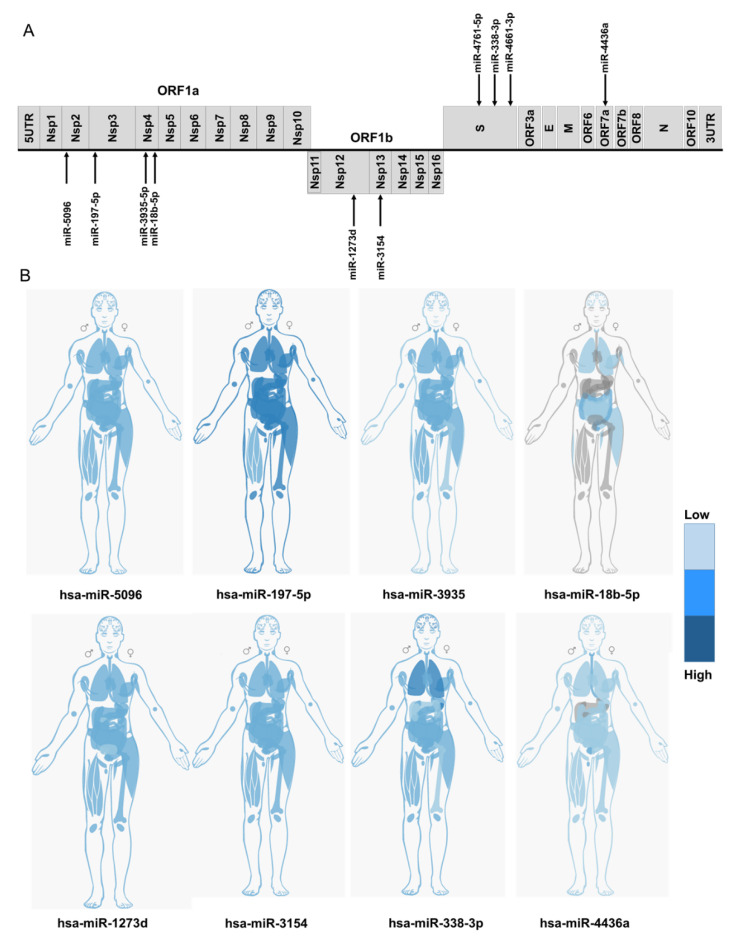
(**A**) Identification of host miRNA targeting different regions of SARS-CoV-2 genome. (**B**) The relative expression level of candidate miRNA in different human tissues. Data was obtained from the IMOTA database. Darker blue indicates the higher expression. Grey colour shows undetectable expression in those tissues. The plotted presentations of miRNA expression in different human tissues obtained from TissueAtlas and TISSUES databases are available in the supplementary figure file.

Three mutations within Nsp4 occur in target sequences of miR-3935 and miR-18b-5p. Both miRNAs are expressed in SAR-CoV-2 target cells (Figure 4B and Figures S7–S10). Nsp4 A9259G is present in a sequence obtained from Vietnam (GISAID: EPI_ISL_416429). Two recurring synonymous mutations, G9802U and G9803U, disrupt the miR-18b binding site of Nsp4. The miR-18b miRNA was reported to be downregulated in viral infections such as HBV and Ebola [57,58] while its expression in patients with cardiovascular disease is upregulated [59,60,61].

**Figure 5:**
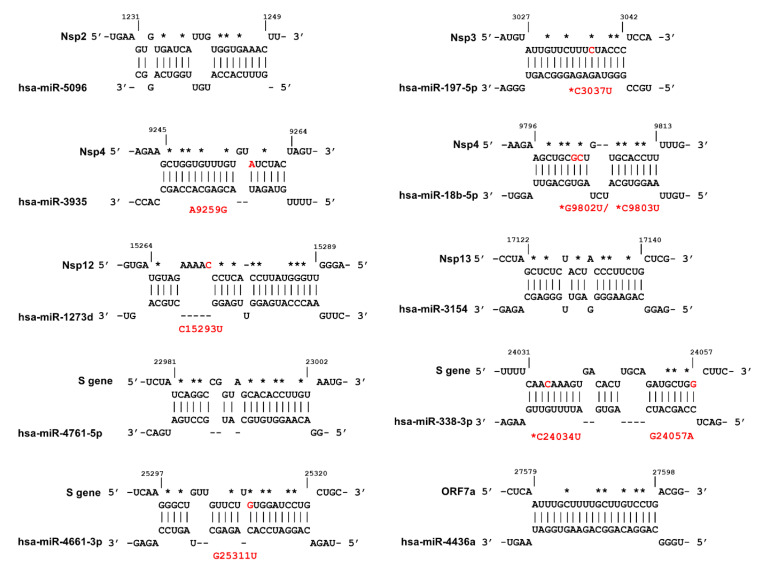
Prediction of host miRNAs binding sites within different regions of SARS-CoV-2 genome. The mutations that occur in miRNA binding sites are indicated in red, and the designations of the mutations are shown in red font. Conserved mutations are indicated with red asterisks while the nucleotide substitutions that result in significant effect on MBS are shown with black asterisks. The figure was produced using IntaRNA tool.

We identified three miRNAs with perfectly matched complementary sequences within the S-gene: miR-338-3p, miR-4661-3p, and miR-4761-5p. As shown in Figure 5, two of these sites were altered by recently identified mutations in the S-gene. In particular, the miR-338-3p miRNA is expressed at high levels in SARS-CoV-2 target cells (Figure 4B, and Figures S14 and S15). The sequences carrying recurrent mutations C24034U and G24057A (EPI_ISL_429691) were predicted to have lost the miR-338-3p binding sites, although these mutations did not decrease the binding energy of miR-338-3p to S (Table S7). The miR-338-3p miRNA acts as a tumour suppressor in liver, lung, and gastric cancers [62,63,64]. The expression level of miR-338-3p declines during HBV infection [65,66] and miR-338-3p has a recognition site within the Vaccinia virus genome [67].

Lastly, G25311U in a patient sample isolated in India (MT396242.1) removed the miR-4661-3p binding site within the S gene (Figure 5, Table S7).

**Figure 6:**
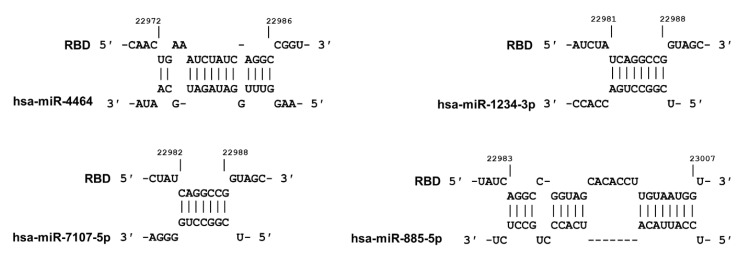
Identification of four miRNAs with ability to bind to the RBD within the S gene. Although not expressed in target cells, these potential miRNAs are included due to their potential use in miRNA-mediated attenuation of the SARS-CoV-2 [37,38].

In addition to the sites mentioned here, we identified an additional four host miRNAs with perfect complementarity within the receptor binding domain (RBD) region of S gene (Figure 6). These miRNAs are not expressed by SARS-CoV-2 target cells (data not shown). However, because these miRNA target sequences exist within the critical ACE-2 targeting region, they may be relevant to miRNA-mediated virus attenuation technology. For example, viral replication can be attenuated in a species-specific and tissue-specific manner by host miRNA machinery, which controls viral tropism, replication, and pathogenesis [35,36,37,38].

### 2.4. Possible Impact of Mutations on Cryptic Splice Sites

Atypical cytoplasmic RNA splicing has been proposed to contribute to non-canonical viral transcripts, even for viruses that classically replicate in the cytoplasm [28,29,30,31,32,33]. Moreover, deep RNA sequencing has identified several previously unidentified SARS-CoV-2 viral RNAs that may be the result of non-canonical splicing events, or alternative transcriptional start sites [34]. We used RegRNA2 [68], HSF [69], and NIPU [70,71] tools to identify the putative splice sites and motifs within the SARS-CoV-2 genome. Our computational prediction identified several 5′ donor and 3′ acceptor splice sites, as well as splice enhancer/inhibitor motifs [72] (Table S8). However, none of the conserved mutations introduced, or deleted, any potential splice sites.

## 3. Discussion

At present there are nearly 200 mutations identified within global SARS-CoV-2 isolates. These mutations are mostly limited to point mutations, with little evidence for recombination events mediating the simultaneous transfer of multiple mutations. Although mutations may be due to RdRP/Nsp12 infidelity, the predominance of C → U and G → A mutations is consistent with base-editing defence (e.g., APOBEC/ADAR) [42,73]. The Nsp14 exonuclease-based proof-reader is a critical counter-defence against host base-editor attack on the coronavirus genome [1]. It is also possible that the position of mutations within the genome could reflect accessibility of host base-editors to the SARS-CoV-2 genome upon uncoating, or during genome translation [42].

In our study, we filtered mutations to common/conserved events according to published sources [39]. There is little evidence that the existing mutations in SARS-CoV-2 have an impact on transmission, replication, or viral load, but our study has flagged potential sites that could impact on viral fitness. It remains to be seen if these mutations be maintained in human populations over time. Carriage of SARS-CoV-2 mutations through rapid expansion into naive populations throughout the world can be due to neutral founder effect, or from fitness gains. However, the ratio of non-synonymous to synonymous mutations is consistent with an emerging virus undergoing purifying selection (see Figure 1 and ref. [40]).

Our study identified a potential binding site for miR-197-5p lost by the Nsp3 synonymous C3037U mutation. miR-197-5p is overexpressed in patients with cardiovascular disease – a patient group that demonstrates an increased susceptibility to SARS-CoV-2 infection. miR-197-5p was previously reported to act in defence against hepatitis viruses, such as HBV, HCV, HAV, and Enterovirus 71 [74,75,76] and was highly elevated in serum of patients with H7N9 [77]. It is possible that a loss of miR-197-5p-mediated defence against SARS-CoV-2 is relevant to the increased mortality noted in this patient group [55]. van Dorp et al. showed that the Nsp3 C3037U mutation was significantly (p = 0.027) associated with ’transmission’—as determined by the relative frequency of sister clades with homoplasies [4]. The C3037U is linked to the A23403G (G614D) mutation [4,56], which may enhance viral infectivity through structural changes in the S protein [5]. Our studies provide further context to monitor the linkage of the C3037U and A23403G sites. However, further investigations into the interactions of miR-197-5p expression, the C3037U mutation, and COVID-19 disease severity in this cardiovascular patients are required. 

It has been shown that folding energy and stability of the mRNA secondary structure influences polypeptide translation and folding. Stable RNA structures act as gauges during translation and reduce the speed of translation to avoid “ribosomal traffic jams” to allow proper folding of newly translated peptides [8]. Therefore, both the sequence and secondary structure of viral mRNA is subject to selection pressure for optimal translation in eukaryotes [9].

Recently, several studies have shown that RNA editing affects the specificity and strength of miRNA binding to its target, and tumour cells may exploit this mechanism to escape from miRNA recognition [78,79]. Three mutations within Nsp4 were predicted to affect miR-3935 and miR-18b-5p targeting. The expression of miR-3935 and miR-18b is altered upon viral infection [57,80,81,82]. The expression level of miR-3935 upregulates during H1N1, Crimean-Congo haemorrhagic fever virus, Coxsackievirus A16, and Enterovirus 71 infection [80,81,82]. The miR-18b was reported to be downregulated during HBV and Ebola virus infections [57,58]. Similar to what was observed for miR-197-5p, both miR-18b and miR-3935 are upregulated in patients with cardiovascular disease [59,60,83]. It should be noted that the effect of total free energy of binding on miRNA function is highly dependent on physiological temperature. For instance, if a mutation increases the ∆G of binding, the effect of mutation will be exacerbated at higher host temperature (e.g., related to the euthermia of the host species, or febrile temperature elevation).

We noted that filtered miRNAs (except miR-338-3p) belong to the GC-rich class of miRNA within their binding region (avg. GC content = 56%). The content of miRNA seed sequence plays critical roles in miRNA function, biogenesis, and ability to downregulate target genes. MiRNAs with higher GC content form relatively more stable duplexes with their target and preferentially originate from canonical pathways of miRNA biogenesis, correlating with greater target suppression [84]. In general, stress-responsive miRNAs have a higher GC content that might enhance miRNA-target duplex stability to activate the stress response [85,86]. Interestingly, the stability of interactions between miRNA and its targets correlates with body temperature: at higher body temperature miRNA-mRNA duplexes with lower GC contents are less functional [85,87]. It should be noted that both 3′ and 5′ ends of miRNAs are responsible for stable and specific interaction between miRNA and its target, particularly if the target region is in a coding region [88,89].

It is not yet clear if anti-viral miRNAs have evolved as host defence against viral infection, or are simply critical gene regulatory elements that assume an additional role for targeting viral transcripts—particularly when the human cellular defence machinery is confronted by an emerging zoonotic virus [13,18,19]. The possibility of including host miRNA binding sites into the genome of live-attenuated viruses offers a further checkpoint for the further attenuation of live vaccines, in a host-cell specific manner. For example, the identification of miRNA target sites in viral pathogens opens up opportunities for further study of viral host cell-tropism, or to create cell-specific or species-specific viral vaccines [35,36,37,38]. Finally, miRNA sites within the coding sequence of viral genes may be critical for ribosomal stalling, leading to the production of pioneer translation products (PTP). Enhanced production of PTP peptides may be critical for MHC-I loading for boosting the anti-viral CTL response [89,90,91,92].

## 4. Methods

### 4.1. Sequence Alignment

The SARS-CoV-2 virus reference sequence was downloaded from NCBI (NC_045512.2) along with 65 sequences up to May 26, 2020 from NCBI or GISAD databases. We included a range of countries with available sequences up to 26 May 2020. In the case of the USA, 16 sequences from 13 states were included. Clustal Omega (using mBed algorithm for guide tree) and Geneious alignment tools were used to perform multiple sequence alignment. The following parameters were used for Geneious alignment: sensitivity; highest/slow, fine tuning; iterate up to five times. Iterative fine tuning involves initial reads to map the consensus sequence, followed by repeated mapping to the consensus sequence. The results are then converted back to mappings relative to the original reference sequence and the process is repeated until the results stabilise, or for a maximum of five iterations.

### 4.2. Mutational Analysis

Mutations with occurrence in multiple sequences originating from different countries were categorized as ‘conserved’. Cumulative plots of the average behaviour of each codon in alignment analysis for insertions/deletions (indels), synonymous (syn), and non-synonymous (nonsyn) substitutions, observed/potential syn and nonsyn mutations, and the ratio of syn to nonsyn substitutions (ds/dn) were calculated using SNAP v2.1.1 for all pairwise comparisons [93]. Natural selection analysis of SARS-CoV-2 sequences in GISAD up to 12th June 2020 was obtained from Observable (https://observablehq.com/).

For mapping the host-spot substitutions which lead to significant change on base pair probabilities of global folding, mode-3 (which is a combination of mode-1/2) of RNAsnp was used. The following parameters were considered using RNAsnp mode-3: folding window—selected size of flanking regions on either side of mutation; 200 nt, *p*-value threshold to filter substitutions that are predicted using mode-2; 0.1, *p*-value threshold to filter substitutions that are predicted using mode-1; 0.05, minimum length of flanking regions on either side of the substitution; 200 nt.

### 4.3. RNA Secondary Structure and Base Pair Probability Analysis

We used well-accepted methods to predict the RNA secondary structure in both wild type and mutated sequences. Minimum free energy (MFE) structures [94] and centroid structures [95] were calculated by RNAfold program to predict RNA secondary structures. To evaluate the impact of mutations on RNA secondary structure and base pair probability, we utilized RNAfold, RNAalifold [96], MutaRNA [71,97], and RNAsnp [98] programs.

The following parameters were used in RNAsnp program: mode-1 (designed to predict the effect of SNPs on short RNA sequences < 1000 bp); folding window (the size of flanking regions on either side of mutation) of 200 nt; minimum length of the sequence interval was 50; cut-off for the base pair probabilities was 0.01. Regardless of the length of sequence, the p values were calculated and presented with both modes (*p* < 0.2 considered significant). MutaRNA was used to calculate the effect of mutations on local folding with a window size of 200 nt and maximal base pair span of 150 nt.

RNAsnp mode-3 and RaSE [71] tools were used to predict the role of each single nucleotide and their substitutions in RNA secondary structure. RaSE program uses EDeN to determine the role of each nucleotide in the RNA secondary structure by assigning a score for each nucleotide based on RNAplfold base pair probabilities. The outputs are: (i) which substitution in each nucleotide has the most effect on RNA structure and (ii) similar to RNAsnp filters, the most significant substitutions. Default parameters were used in the RaSE structure graph, RNAplfold, and EDeN.

### 4.4. Potential miRNA Binding Site Analysis

For identifying potential miRNA binding sites, the SARS-COV-2 genome was screened with RegRNA2 (filtered to human miRNAs, score ≥ 170, free energy ≤ −25) and miRDB (custom prediction tool) [99]. We excluded miRNAs not expressed in SARS-CoV-2 target cells such as lung, oesophagus, kidney, and small intestine [100,101]. The expression levels of miRNA in target cells were determined by TissueAtlas [102], IMOTA [103], TISSUES [104], or using published data. The impact of mutations on miRNA binding was visualized by RegRNA2.0, miRDB, IntaRNA (one interaction per RNA pair, minimum 7 base pairs in seed, no seed with GU end, no lonely base pairs) [105] and CopomuS (no A:U, G:U base pairs, no lonely base pairs, no helix ends, IntaRNA parameters: no GU at helix ends, min. 7 base pairs in seed) [71], and RNAup (avoid isolated base pairs, length of the unstructured region; 4nt, maximal length of the region of interaction; 25nt). We used IntaRNA to illustrate miRNA binding to its target.

Wild type and mutated sequences were analysed by RegRNA2.0 and miRDB to determine if mutations result in a loss of miRNA binding prediction. In addition, the total free energy of binding (∆G) was calculated with IntaRNA and RNAup. If WT ∆G < Mut ∆G, the mutation was assumed to reduce the strength of miRNA binding to the target sequence.

### 4.5. Potential Splice Site Analysis

Potential splice donor/acceptor splice sites, exon splicing enhancer (ESE), exon splicing silencer (ESS), intron splicing enhancer (ISE), and intron splicing silencer (ISS) motifs were predicted using RegRNA2.0 [68], HSF [69], and NIPU [70,71] tools.

## Figures and Tables

**Table 1:** Conserved mutations in SARS-CoV-2 genome.

Gene	Mutation	Amino Acid Change
**5′ UTR**	C to U—nt241	-
**Nsp1**	C to U—nt313	No (L16)
**Nsp2**	C to U—nt1059	T85I
	G to A—nt1397	V198I
	Deletion 1606–1609	D268 deletion
**Nsp3**	C to U—nt3037	No (F106)
**Nsp4**	C to U—nt8782	No (S76)
	C to U—9802	No (A416)
	G to U—9803	No (L417)
**Nsp6**	G to U—nt11083	L37F
**Nsp12**	C to U—nt14408	P232L
	C to U—nt14805	No (Y455)
**Nsp13**	U to C—nt17247	No (R337)
**S**	A to G—nt23403	D614G
	C to U—nt24034	No (N824)
**ORF3a**	G to U—nt25563	Q57H
	G to U—nt26144	G251V
**ORF8**	C to U—nt27964	S24L
	U to C- nt28144	L84S
**N**	C to U—nt28311	P13L
	U to C—nt28688	No (L139)
	GGG to AAC—nt28881-28884	R203K and G204R
**3′ UTR**	G to U—nt29742	-

## Supplementary Materials

The following are available online at https://www.mdpi.com/1422-0067/21/13/4807/s1.
