# Supplementary material for: Implications of SARS-CoV-2 Mutations for Genomic RNA Structure and Host microRNA Targeting
Source: Int J Mol Sci. 2020 Jul 7;21(13):4807. doi: 10.3390/ijms21134807 (PMC7370282; doi:10.3390/ijms21134807)
Supplement: Supplementary file 1 [file ijms-21-04807-s001.zip › Supplemenatry Data-Tables.pdf]

Table S1. Sequence alignment and identification of recurrent mutations identified in the SARS-CoV-2 genome.

	Sequence identifiers	% Pairwise identity	Country	Conserved mutation(s)
Ref	NC_045512.2		China	
1	MT276598.1	99.97	Israel	C241U/C313U/C3037U/C14408U/A23403G/GGG28881-4AAC
2	MT066176.1	99.99	Taiwan	
3	MT262993.1	99.9	Pakistan	
4	MT344962.1	99.98	USA/Minnesota	Del 1606-1609
5	MT344959.1	99.98	USA/Pennsylvania	C241U/C1059U/C3037U/C14408U/A23403G/G25563U
6	MT304476.1	99.98	South Korea	G11083U/C14805U/G26144U/C28311U
7	MT276326.1	99.98	USA/Georgia	G11083U/C14805U/G26144U
8	MT320538.2	99.98	France	C241U/C3037U/C14408U/A23403G
9	MT344960.1	99.98	USA/Rhode Island	C241U/C1059U/C3037U/C14408U/A23403G/G25563U/C27964U
10	MT344961.1	99.98	USA/Nevada	Del 1606-1609
11	MT328034.1	99.97	Greece	C8782U/C14805U/ U28134C
12	MT093571.1	99.98	Sweden	G26144U
13	MT114415.1	99.99	Hong Kong	G1397A/G11083U/U28688C/G29742U
14	MT350282.1	99.98	Brazil	G11083U/C14408U/C14805U/U17247C/G26144U
15	MT470156.1	99.97	France	C241U/C313U/C3037U/A23403G/GGG28881-4AAC
16	MT328035.1	99.97	Greece	C241U/C3037U/C14408U/A23403G/GGG28881-4AAC
17	MT371050.1	99.99	Sri Lanka	C14805U/U17247C/G26144U
18	MT370518.1	99.98	Taiwan	C1059U/G1397A/G11083U/U28688C/G29742U
19	LC542976.1	99.99	Japan	C8782U/ U28134C
20	MT371047.1	99.97	Sir Lanka	G1397A/G11083U/U28688C/G29742U
21	MT324062.1	99.98	South Africa	C241U/C3037U/C14408U/A23403G
22	MT396241.1	99.99	China	
23	MT007544.1	99.96	Australia	G26144U
24	MT385418.1	99.97	USA/California	C241U/C313U/C3037U/C14408U/A23403G/GGG28881-4AAC
25	MT374105.1	99.99	Taiwan	G11083U/C14805U/U17247C/G26144U
26	MT374116.1	99.97	Taiwan	C241U/C3037U/C14408U/A23403G/GGG28881-4AAC
27	MT327745.1	99.98	Turkey	G1397A/G11083U/U28688C/G29742U
28	MT358402.1	99.97	USA/Louisiana	C241U/C1059U/C3037U/C14408U/A23403G/G25563U/C27964U
29	MT263074.1	99.97	Peru	C241U/C3037U/C14408U/A23403G/GGG28881-4AAC
30	MT359866.1	99.97	Spain	C241U/C313U/C3037U/C14408U/A23403G/GGG28881-4AAC
31	MT385486.1	98.6	USA/California	G11083U/C28311U
32	MT072688.1	99.99	Nepal	C24023U
33	LC553263.1	99.97	Japan	C241U/C313U/C3037U/C14408U/A23403G/GGG28881-4AAC
34	MT114419.1	99.99	Hong Kong	G11083U
35	MT396242.1	99.5	India	C241U/C313U/C3037U/C14408U/A23403G/GGG28881-4AAC
36	MT375478.1	99.97	USA/Washington	C241U/C1059U/C3037U/C14408U/A23403G/G25563U
37	MT240479.1	99.98	Pakistan	C241U/G1397A/G11083U
38	MT372482.1	99.1	Malaysia	G11083U/C28311U
39	MT350242.1	99.9	USA/Virginia	C241U/C1059U/C14408U/A23403G/G25563U
40	MT320891.2	99.98	Iran	G1397A/G11083U/G29742U
41	MT198651.1	96.4	Spain	C8782U/C14805U/U28134C
42	MT292575.1	99.98	Spain	C241U/C3037U/C14408U/A23403G
43	MT582453.1	99.97	Germany	C241U/C1059U/C3037U/C14408U/A23403G/G25563U
44	MT371035.1	99.97	USA/New York	C241U/C1059U/C3037U/C14408U/A23403G/G25563U
45	MT370841.1	99.96	USA/New York	C8782U/A23403G/C24023U/U28134C
46	MT077125.1	99.99	Italy	G11083U/G26144U
47	MT370933.1	99.98	USA/New Jersey	C241U/C1059U/C3037U/C14408U/A23403G/G25563U
48	MT358731.1	99.97	USA/Washington	C241U/C1059U/C3037U/C14408U/A23403G/G25563U
49	MT371574.1	99.98	Czech Republic	C241U/C3037U/C14408U/A23403G/GGG28881-4AAC
50	MT375471.1	99.98	USA/Connecticut	C1059U/C3037U/C14408U/A23403G/G25563U
51	MT451006.1	99.99	Australia	Del 1606-1609
52	MT457394.1	99.95	Nederland	Del 1606-1609
53	MT451263.1	99.5	Australia	C241U/C3037U/G9802U/C14408U/A23403G
54	MT496988.1	99.98	India	C241U/C3037U/G9802U/C14408U/A23403G/G25563U
55	EPI_ISL_416425	99.9	China	G9802U
56	EPI_ISL_417211	99.98	New Zealand	Del 1606-1609
57	EPI_ISL_416429	99.98	Vietnam	C8782U/C24023U/U28134C
58	EPI_ISL_419222	99.6	Hong Kong	G26144U
59	EPI_ISL_422940	99.5	Netherlands	C241U/C3037U/G9803U/C14408U/A23403G/GGG28881-4AAC/
60	MT451063.1	99.3	Australia	C8781U/G9803U
61	MT380727.1	99.9	Iran	U28688C
62	MT512430.1	99.99	USA/Atlanta	G9803U
63	EPI_ISL_417212	99.97	New Zealand	C14805U/G26144U
64	EPI_ISL_429691	90.2	Brazil	C3037U/C14408U/A23403G/GGG28881-4AAC
65	EPI_ISL_416538	99.6	New Zealand	C8781U/C24034U

Table S2. Mutational analysis of SAR-CoV-2 sequences analyzed in the current study.

Sequence identifiers	Sd	Sn	S	N	ps	pn	ds	dn	ds/dn	ps/pn
China-EPI_ISL_416425	5.0000	5.0000	6559.3333	23341.6667	0.0008	0.0002	0.0008	0.0002	3.5598	3.5585
Israel-MT276598.1	5.0000	3.0000	6556.0000	23309.0000	0.0008	0.0001	0.0008	0.0001	5.9281	5.9256
Taiwan-MT066176.1	1.0000	1.0000	6556.3333	23311.6667	0.0002	0.0000	0.0002	0.0000	3.5559	3.5556
Vietnam-EPI_ISL_416429	4.0000	3.0000	6556.6667	23311.3333	0.0006	0.0001	0.0006	0.0001	4.7420	4.7405
USA-MT344962.1	2.0000	0.0000	6556.6667	23314.3333	0.0003	0.0000	0.0003	0.0000	nan	nan
USA-MT512430.1	1.0000	0.0000	6557.3333	23322.6667	0.0002	0.0000	0.0002	0.0000	nan	nan
France-MT320538.2	3.0000	3.0000	6557.5000	23322.5000	0.0005	0.0001	0.0005	0.0001	3.5574	3.5566
USA-MT276326.1	1.0000	5.0000	6557.3333	23322.6667	0.0002	0.0002	0.0002	0.0002	0.7113	0.7113
USA-MT344960.1	4.0000	3.0000	6557.5000	23322.5000	0.0006	0.0001	0.0006	0.0001	4.7437	4.7422
USA-MT344959.1	3.0000	3.0000	6556.5000	23320.5000	0.0005	0.0001	0.0005	0.0001	3.5576	3.5569
USA-MT344961.1	1.0000	1.0000	6556.5000	23317.5000	0.0002	0.0000	0.0002	0.0000	3.5567	3.5564
South_Korea-MT304476.1	0.0000	5.0000	6557.3333	23322.6667	0.0000	0.0002	0.0000	0.0002	nan	nan
Greece-MT328034.1	4.0000	5.0000	6557.8333	23325.1667	0.0006	0.0002	0.0006	0.0002	2.8462	2.8455
Sweden-MT093571.1	2.0000	5.0000	6558.1667	23324.8333	0.0003	0.0002	0.0003	0.0002	1.4227	1.4226
Hong Kong-MT114415.1	1.0000	3.0000	6558.6667	23330.3333	0.0002	0.0001	0.0002	0.0001	1.1857	1.1857
Brazil-MT350282.1	0.0000	6.0000	6559.1667	23341.8333	0.0000	0.0003	0.0000	0.0003	nan	nan
France-MT470156.1	5.0000	3.0000	6560.0000	23341.0000	0.0008	0.0001	0.0008	0.0001	5.9326	5.9301
Greece-MT328035.1	4.0000	4.0000	6559.8333	23341.1667	0.0006	0.0002	0.0006	0.0002	3.5592	3.5582
Hong Kong-EPI_ISL_419222	1.0000	3.0000	6537.0000	23244.0000	0.0002	0.0001	0.0002	0.0001	1.1853	1.1853
Japan-LC542976.1	4.0000	0.0000	6559.3333	23341.6667	0.0006	0.0000	0.0006	0.0000	nan	nan
NewZealand-EPI_ISL_416538	4.0000	7.0000	6537.8333	23243.1667	0.0006	0.0003	0.0006	0.0003	2.0320	2.0315
Sir Lanka-MT371047.1	4.0000	6.0000	6560.1667	23340.8333	0.0006	0.0003	0.0006	0.0003	2.3725	2.3720
South Africa-MT324062.1	4.0000	2.0000	6559.5000	23341.5000	0.0006	0.0001	0.0006	0.0001	7.1193	7.1169
Taiwan-MT370518.1	2.0000	5.0000	6559.6667	23341.3333	0.0003	0.0002	0.0003	0.0002	1.4234	1.4233
Sri Lanka_-MT371050.1	1.0000	3.0000	6559.5000	23341.5000	0.0002	0.0001	0.0002	0.0001	1.1862	1.1861
China-MT396241.1	1.0000	0.0000	6559.3333	23335.6667	0.0002	0.0000	0.0002	0.0000	nan	nan
Australia-MT007544.1	0.0000	3.0000	6556.1667	23332.8333	0.0000	0.0001	0.0000	0.0001	nan	nan
USA-MT385418.1	5.0000	4.0000	6560.0000	23341.0000	0.0008	0.0002	0.0008	0.0002	4.4494	4.4476
Taiwan-MT374105.1	0.0000	4.0000	6558.6667	23339.3333	0.0000	0.0002	0.0000	0.0002	nan	nan
Taiwan-MT374116.1	5.0000	3.0000	6559.3333	23338.6667	0.0008	0.0001	0.0008	0.0001	5.9326	5.9301
Turkey-MT327745.1	2.0000	4.0000	6550.1667	23281.8333	0.0003	0.0002	0.0003	0.0002	1.7774	1.7772
USA-MT358402.1	5.0000	5.0000	6558.6667	23336.3333	0.0008	0.0002	0.0008	0.0002	3.5594	3.5581
Peru-MT263074.1	5.0000	4.0000	6553.6667	23299.3333	0.0008	0.0002	0.0008	0.0002	4.4457	4.4439
Nederland-MT457394.1	5.0000	6.0000	6556.0000	23318.0000	0.0008	0.0003	0.0008	0.0003	2.9650	2.9640
Spain-MT359866.1	5.0000	4.0000	6558.0000	23334.0000	0.0008	0.0002	0.0008	0.0002	4.4494	4.4476
USA-MT385486.1	3.0000	4.0000	6466.3333	23002.6667	0.0005	0.0002	0.0005	0.0002	2.6685	2.6680
Nepal-MT072688.1	1.0000	0.0000	6543.3333	23264.6667	0.0002	0.0000	0.0002	0.0000	nan	nan
Japan-LC553263.1	6.0000	4.0000	6556.3333	23326.6667	0.0009	0.0002	0.0009	0.0002	5.3395	5.3368
Hong-Kong-MT114419.1	1.0000	1.0000	6552.5000	23309.5000	0.0002	0.0000	0.0002	0.0000	3.5576	3.5573
India-MT396242.1	5.0000	8.0000	6511.5000	23149.5000	0.0008	0.0003	0.0008	0.0003	2.2226	2.2220
USA-MT375478.1	3.0000	6.0000	6552.0000	23316.0000	0.0005	0.0003	0.0005	0.0003	1.7795	1.7793
Pakistan-MT240479.1	2.0000	4.0000	6547.6667	23287.3333	0.0003	0.0002	0.0003	0.0002	1.7785	1.7783
Australia-MT451263.1	4.0000	2.0000	6448.8333	22987.1667	0.0006	0.0001	0.0006	0.0001	7.1316	7.1291
USA-MT350242.1	3.0000	4.0000	6548.8333	23301.1667	0.0005	0.0002	0.0005	0.0002	2.6691	2.6685
Malaysia-MT372482.1	6.5000	30.5000	6488.8333	23121.1667	0.0010	0.0013	0.0010	0.0013	0.7592	0.7594
Australia-MT451006.1	0.0000	1.0000	6543.0000	23253.0000	0.0000	0.0000	0.0000	0.0000	nan	nan
India-MT496988.1	6.0000	4.0000	6543.1667	23255.8333	0.0009	0.0002	0.0009	0.0002	5.3340	5.3313
Iran-MT320891.2	1.0000	4.0000	6545.3333	23274.6667	0.0002	0.0002	0.0002	0.0002	0.8890	0.8890
Australia-MT451063.1	5.0000	2.0000	6473.8333	23025.1667	0.0008	0.0001	0.0008	0.0001	8.8957	8.8916
Spain-MT198651.1	3.0000	5.0000	6155.0000	21958.0000	0.0005	0.0002	0.0005	0.0002	2.1409	2.1405
Netherlands-EPI_ISL_422940	6.0000	4.0000	6492.0000	23100.0000	0.0009	0.0002	0.0009	0.0002	5.3400	5.3373
Spain-MT292575.1	3.0000	2.0000	6536.5000	23238.5000	0.0005	0.0001	0.0005	0.0001	5.3341	5.3328
Germany-MT394864.1	3.0000	3.0000	6284.1667	22371.8333	0.0005	0.0001	0.0005	0.0001	3.5608	3.5600
Germany-MT582453.1	3.0000	4.0000	6536.8333	23238.1667	0.0005	0.0002	0.0005	0.0002	2.6667	2.6662
USA-NY-MT371035.1	3.0000	7.0000	6524.3333	23199.6667	0.0005	0.0003	0.0005	0.0003	1.5241	1.5239
USA-MT370841.1	5.0000	7.0000	6524.8333	23199.1667	0.0008	0.0003	0.0008	0.0003	2.5404	2.5397
Italy-MT077125.1	0.0000	2.0000	6537.8333	23243.1667	0.0000	0.0001	0.0000	0.0001	nan	nan
USA-MT370933.1	3.0000	3.0000	6522.1667	23192.8333	0.0005	0.0001	0.0005	0.0001	3.5568	3.5560
USA-MT358731.1	4.0000	4.0000	6534.5000	23267.5000	0.0006	0.0002	0.0006	0.0002	3.5618	3.5607
Czech Republic-MT371574.1	4.0000	3.0000	6529.0000	23225.0000	0.0006	0.0001	0.0006	0.0001	4.7445	4.7429
NewZealand-EPI_ISL_417211	2.0000	0.0000	6513.0000	23166.0000	0.0003	0.0000	0.0003	0.0000	nan	nan
Brazil-EPI_ISL_429691	4.0000	9.0000	5614.5000	20026.5000	0.0007	0.0004	0.0007	0.0004	1.5856	1.5853
NewZealand-EPI_ISL_417212	1.0000	6.0000	6511.3333	23170.6667	0.0002	0.0003	0.0002	0.0003	0.5930	0.5931
USA-MT375471.1	2.0000	5.0000	6491.6667	23151.3333	0.0003	0.0002	0.0003	0.0002	1.4266	1.4265
Iran-MT380727.1	1.0000	0.0000	300.3333	1016.6667	0.0033	0.0000	0.0033	0.0000	nan	nan

Sd: the number of observed synonymous substitutions / Sn: The number of observed non-synonymous substitutions / S: The number of potential synonymous substitutions (the average for the two compared sequences) / N: The number of potential non-synonymous substitutions (the average for the two compared sequences) ps: The proportion of observed synonymous substitutions (Sd/S) / pn: The proportion of observed non-synonymous substitutions (Sn/N) / dS: The Jukes-Cantor correction for multiple hits of ps / dN: The Jukes-Cantor correction for multiple hits of pn/ ds/dn: The ratio of synonymous to non-synonymous substitutions. Note, the total number of mutations in a virus sequence is the sum of synonymous and non-synonymous substitutions (Sd+Sn).

Table S3. Nucleotide substitutions that have the greatest impact on RNA secondary structure in the 200 bp surrounding region of 1059 position in Nsp2 based on RNAsnp mode-3.

Nsp2	Mode 2		Mode 1		
**Mutation**	**Interval**	**p-value1**	**Folding Window**	**Local region**	**p-value2**
G960A	20-50	0.0975	1-200	1-50	0.0467
G960C	1-33	0.0047	1-200	1-50	0.0297
G960U	20-50	0.0962	1-200	1-50	0.0395
U961G	1-31	0.0238	1-200	132-199	0.0282
G970C	1-31	0.0010	1-200	1-50	0.0379
G1018U	53-125	0.0163	1-200	55-124	0.0299
A1020C	56-125	0.0060	1-200	57-125	0.0203
A1022C	61-124	0.0134	1-200	62-124	0.0331
C1026G	30-71	0.0097	1-200	32-125	0.0384
U1029C	59-125	0.0198	1-200	55-124	0.0372
A1032C	53-125	0.0087	1-200	55-124	0.0267
A1032U	53-125	0.0088	1-200	55-124	0.0269
A1037C	52-125	0.0117	1-200	53-125	0.0313
A1037U	52-125	0.0157	1-200	53-125	0.0394
A1038C	52-125	0.0136	1-200	52-125	0.0383
C1044G	77-134	0.0093	1-200	62-134	0.0211
A1047C	78-127	0.0195	1-200	60-127	0.0335
A1051G	52-106	0.0108	1-200	53-106	0.0334
U1053C	52-106	0.0067	1-200	53-106	0.0248
U1054G	61-120	0.0127	1-200	62-120	0.0381
U1062G	55-110	0.0117	1-200	56-110	0.0343
U1062C	61-124	0.0168	1-200	62-124	0.0449
A1064C	61-124	0.0168	1-200	62-124	0.0471
U1073G	83-124	0.0170	1-200	62-124	0.0265
U1086G	99-135	0.0132	1-200	53-135	0.0480
U1090G	77-133	0.0246	1-200	62-133	0.0439
C1091G	77-134	0.0074	1-200	62-134	0.0224
C1121G	142-198	0.0022	1-200	142-198	0.0126

Table S4. Nucleotide substitutions that have the most impact on RNA secondary structure on the 3′UTR based on RNAsnp mode-3.

3′ UTR	Mode 2		Mode 1		
**Mutation**	**Interval**	**p-value1**	**Folding Window**	**Local region**	**p-value2**
A29700C	15-99	0.0860	1-226	11-93	0.0306
G297003U	15-99	0.0813	1-229	11-93	0.0406
U29709C	15-46	0.0446	1-229	1-50	0.0431
A29749C	15-99	0.0886	1-229	11-93	0.0224

Table S5. Nucleotide substitutions that have the greatest impact on RNA secondary structure on 3′UTR based on RaSE program. The dot-bracket-notation of MFE structure of mutated sequence is presented.

Position	WT	Mut	Similarity	MFE
29695	A	G	0.71	((.((((((((((((.((((((((((..((.(((....))))).)).((((((((...(..(((.(((.......))).)))..).))))).)))).(((((....((.((.....)).))..)))))..................)))))))))...))).)))))..............))))).)).................................
29810	G	C	0.71	...((((((((..((.....))))))))))((.(((....)))))....(((((((.(((.(....).))).(((((......)))))...........((((((((.......(((((((...(((.(((.....((((((....))))))))).)))....)))))))..........))))))))))))))...................................
29844	A	G	0.71	...((((((((..((.....))))))))))((.(((....)))))....(((((((.(((.(....).))).(((((......)))))...........((((((((....(((.((((((...(((.(((.((..............)).))).)))....)))))))))........)))))))))))))).................................
29697	A	C	0.72	((.((((...((.(((((...((((.(((((((..((..........))..))))).)))))).))))).))(((((......)))))...........((((((((.......(((((((...(((.(((.((..............)).))).)))....))))))).........))))))))))).)).................................
29716	A	U	0.72	...........(((((...))))).(((((.(((((((((((......))))))))(((.(....).))).(((((......)))))......(((....(((((....((.((.....)).))..)))))...)))...............))).)..))))..(((.(((((..(((....)))..))))).)))...............................
29749	A	U	0.72	...((((((((..((.....))))))))))((.(((....)))))....(((((((...(((((.....(((((((..((.(((((..........))))).))..))))).))(((((((...(((.(((.((..............)).))).)))....)))))))).........)))))....)))))))..................................
29806	A	U	0.72	...((((((((..((.....))))))))))((.(((....)))))....(((((((.(((.(....).))).(((((......)))))...........((((((((.......(((((((...(((.((((((..............)))))).)))....)))))))..........)))))))))))))).................................
29809	U	G	0.72	...((((((((..((.....))))))))))((.(((....)))))....(((((((...(((((((((.((.(((((......)))))............(((((....((.((.....)).))..)))))..(((((..((((.......))))..))))))).))))..........)))))....))))))).................................
29853	G	U	0.72	...((((((((..((.....))))))))))((.(((....)))))....(((((((.(((.(....).))).(((((......)))))...........(((((((((......(((((((...(((.(((.((..............)).))).)))....)))))))..........))))))))))))).................................
29863	A	G	0.72	....((..(((.(((.....((((((((.((.(((....)))))....((((((((...(..(((.(((.......))).)))..).))))).)))).((((........))))..........))))))))......))).)))..))..((....((((.((.((...........)).)))))))...))...............................

Table S6. Nucleotide substitutions that have the greatest impact on RNA secondary structure of 200 bp flanking region of 1059 nucleotide in Nsp2 based on RaSE program. The dot-bracket-notation of MFE structure of mutated sequence is presented.

Position	WT	Mut	Similarity	MFE
970	G	C	0.45	..........((((((.....((((((....((((.(((((....)))))...(((.((((((((((.(((((.(((((((........)))))))....))))).)))...))))).......)))).)))).......))))...)))))).((.(((((((...))))))).))....((((....)))))))))..
980	G	U	0.48	...................(((((((....((((.(((((....)))))...(((.((((((((((.(((((.(((((((........)))))))....))))).)))...)))).......)))).)))........))))...)))))))((.((((((...))))))).))..((((((....))))))......
961	U	G	0.50	(((((...(((((......)))))...........(((((....)))))((((((.....(((((((.(((((.(((((((........)))))))....))))).)))...))))......)))))).)))))......((((((.......((.(((((((...))))))).))...(((((....)))))))))))).
989	A	C	0.53	....((..(((((......))))).))((((((((.(((((....)))))...(((.((((((((((.(((((.(((((((........)))))))....))))).)))...))))........)))).))).......))))..........((.(((((((...))))))).))..((((((....))))))..))))
1000	U	C	0.53	(((.((..(((((......))))).)).)))(((.((((((.......((........)).....((..(((((.(((((((........)))))))....))))).)))))))))))............(((.((((..(((...(((((.((.((((((...))))))).))......))))))).)))).))).
1091	C	G	0.53	(((.((..(((((......))))).)).)))((((.(((((....)))))....((((.((((.....)))).))))......((((((((((((((((((((....))))..))).))))))))...))))))......))))..........((.((((((...))))))).))..((((((....))))))......
1006	G	A	0.54	(((.((..(((((......))))).)).)))(((.(((((........((........)).....(((.(((((.(((((((........)))))))....))))).))))))))))))............(((.((((..(((...(((((.((.((((((...))))))).))......))))))).)))).))).
1044	C	G	0.54	(((.((..(((((......))))).)).)))((((.(((((....)))))....((((.((((.....)))).))))......((((((((((((((((((((....))))..))).))))))))...))))))......))))..........((.((((((...))))))).))..((((((....))))))......
1060	C	G	0.54	(((.((..(((((......))))).)).)))((((.(((((....)))))..((((((((((....(((((..(.......)..)))))...)))).)).))))))..((((((((.(.........)))))))))......))))..........((.(((((((...))))))).))..((((((....))))))......
1073	U	C	0.54	(((.((..(((((......))))).)).)))(((.(((((........((........)).....(((.(((((.(((((((........)))))))....))))).))))))))))))............(((.((((..(((...(((((.((.((((((...))))))).))......))))))).)))).))).

Table S7. The effect of mutations in SARS-CoV-2 genes on prediction of host miRNA binding and total free energy of binding miRNA binding using ItaRNA and RNAup tools. Conserved mutations are indicated with asterisks.

**Target**		**Predicted miRNA**	**Total free energy of binding**	
			IntaRNA	RNAup
**Nsp3**	WT	miR-197-5p	-12.24	-13.37
	*C3037U	-	-8.53	-9.75
**Nsp4**	WT	miR-3935	-13.39	-14.73
	A9259G	-	-13.48	-14.78
**Nsp4**	WT	miR-18b-5p	-13.72	-13.5
	*G9802U	-	-10.13	-9.6
	*C9803U	-	-8.7	-8.85
**Nsp12**	WT	miR-1273d	-16.42	-16.61
	C15293U	miR-1273d	-15.22	-15.48
**S**	WT	miR-338-3p	-6.98	-6.54
	C24034U	-	-6.98	-6.54
	G24057A	-	-8	-7.83
**S**	WT	miR-4661-3p	-19.2	-14.41
	G25311U	-	-17.23	-13.14

Table S8. Putative donor and acceptor splice sites and splice motifs in SARS-CoV-2 genome.

Region	5′ Donor site	3′ Acceptor site	ESE	ESS	ISE	ISS
5′ UTR	-	1	-	-	-	-
Nsp1	2	-	1	1	8	-
Nsp2	1	1	3	2	6	1
Nsp3	3	3	25	12	25	-
Nsp4	4	-	6	1	6	-
Nsp5	2	1	3	3	6	-
Nsp6	-	3	-	1	5	-
Nsp7	-	-	2	2	-	-
Nsp8	1	-	5	1	2	-
Nsp9	1	-	2	-	2	-
Nsp10	1	-	2	-	1	-
Nsp11	-	-	-	-	1	-
Nsp12	1	5	9	7	10	-
Nsp13	2	2	5	1	9	-
Nsp14	1	3	4	1	7	-
Nsp15	-	-	1	1	-	-
Nsp16	-	1	1	1	-	-
S	-	6	6	3	14	1
ORF3a	-	3	4	3	3	-
E	1	1	-	-	4	-
M	-	1	1	2	5	-
ORF6	-	-	-	1	1	-
ORF7a	-	1	1	-	3	-
ORF7b	-	-	-	-	-	-
ORF8	1	-	1	-	1	-
N	2	3	7	7	6	-
ORF10	-	-	-	-	-	-
3′ UTR	-	-	-	1	-	-

ESE; exon splicing enhancer, ESS; exon splicing silencer, ISE; intron splicing enhancer, ISS; intron splicing silencer.
