# Supplementary material for: Implications of SARS-CoV-2 Mutations for Genomic RNA Structure and Host microRNA Targeting
Source: Int J Mol Sci. 2020 Jul 7;21(13):4807. doi: 10.3390/ijms21134807 (PMC7370282; doi:10.3390/ijms21134807)
Supplement: Supplementary file 1 [file ijms-21-04807-s001.zip › Supplemenatry Figures.pdf]

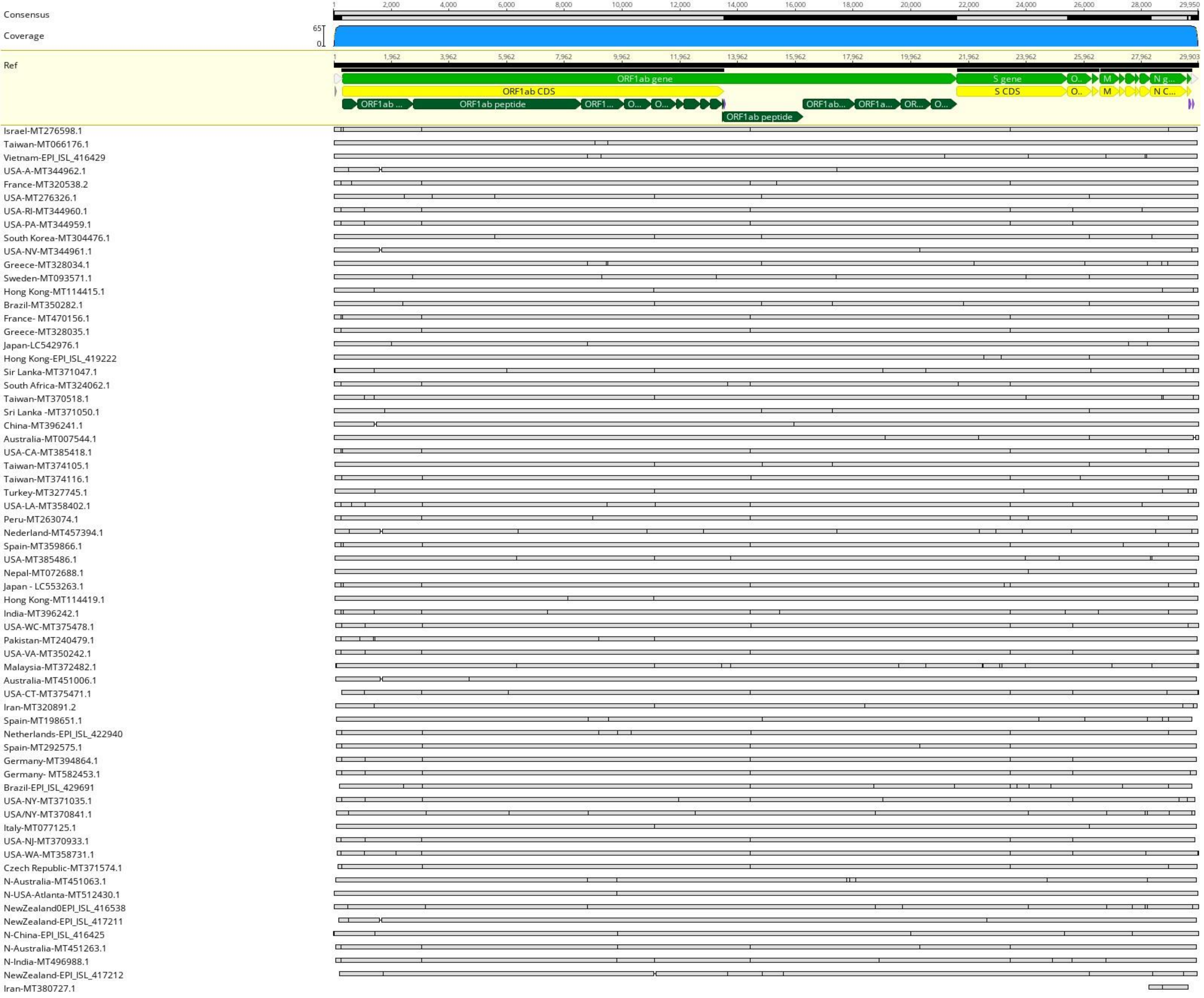

Figure S1. An overview of Geneious alignment of the analyzed sequences mapped to the reference sequence. Mutations are indicated in black vertical lines.

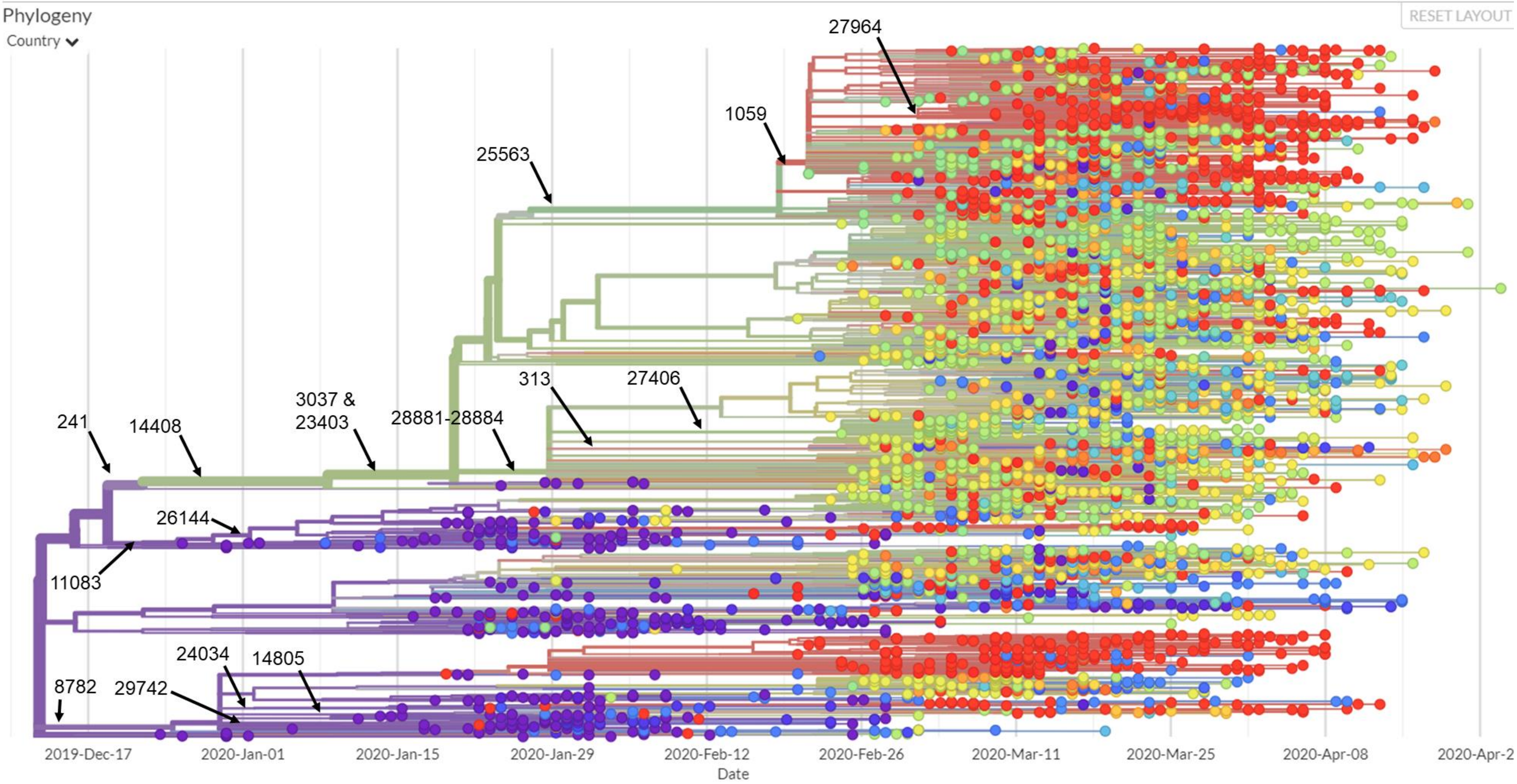

Figure S2. The position of selected, conserved mutations studied here are illustrated in phylogeny graph to demonstrate that the majority of the mutations under study are conserved via founder effect. Note, due to the density of the original GASAID graph only mutations that could be precisely positioned on the graph are illustrated. Modified from the Nextstrain database. Picture captured on May 1st , 11: 21 AM.

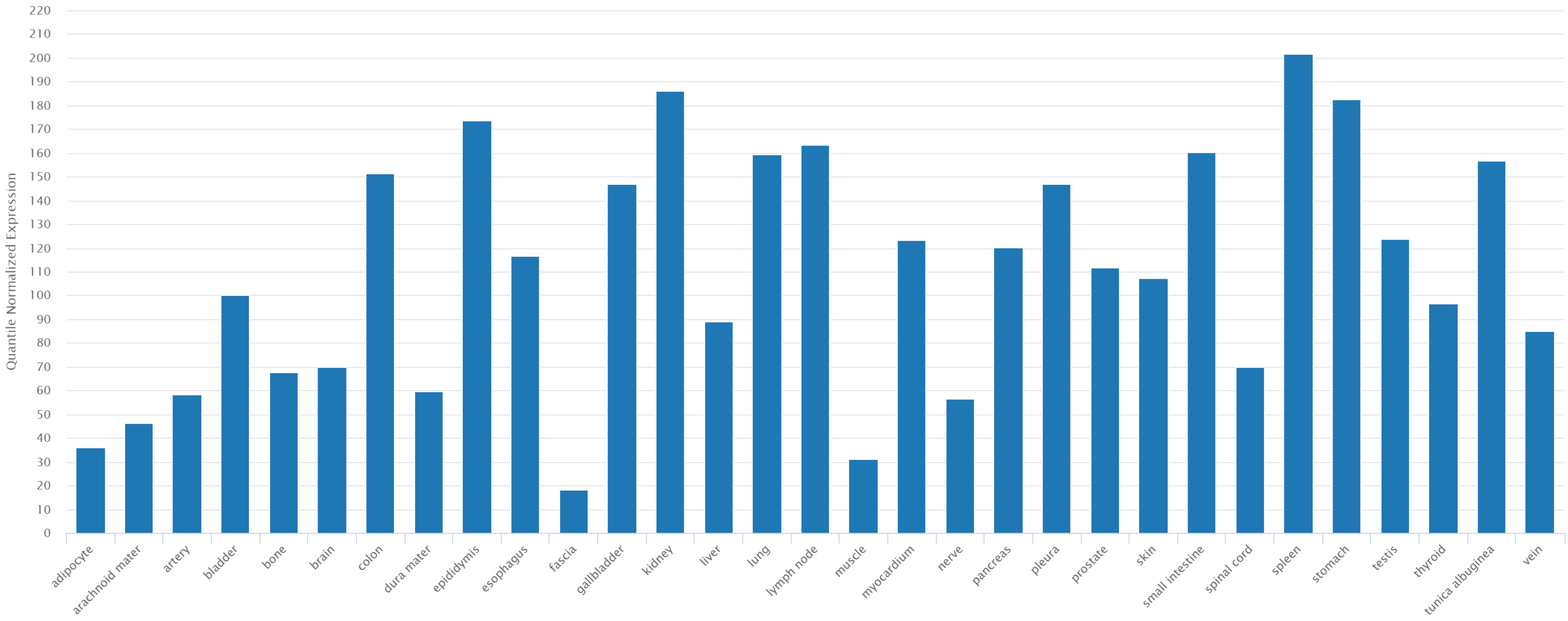

Figure S3. The expression of miR-5096 in different human tissue obtained from TissueAtlas data base. The graph represents expression level in two different samples.

hsa-miR-5096 tissues

hsa-miR-5096 [hsa-miR-5096]

Synonyms: hsa-miR-5096, mir-5096, MIMAT0020603, MI0018004, hsa-mir-5096 ...

Linkouts: STRING Pharos

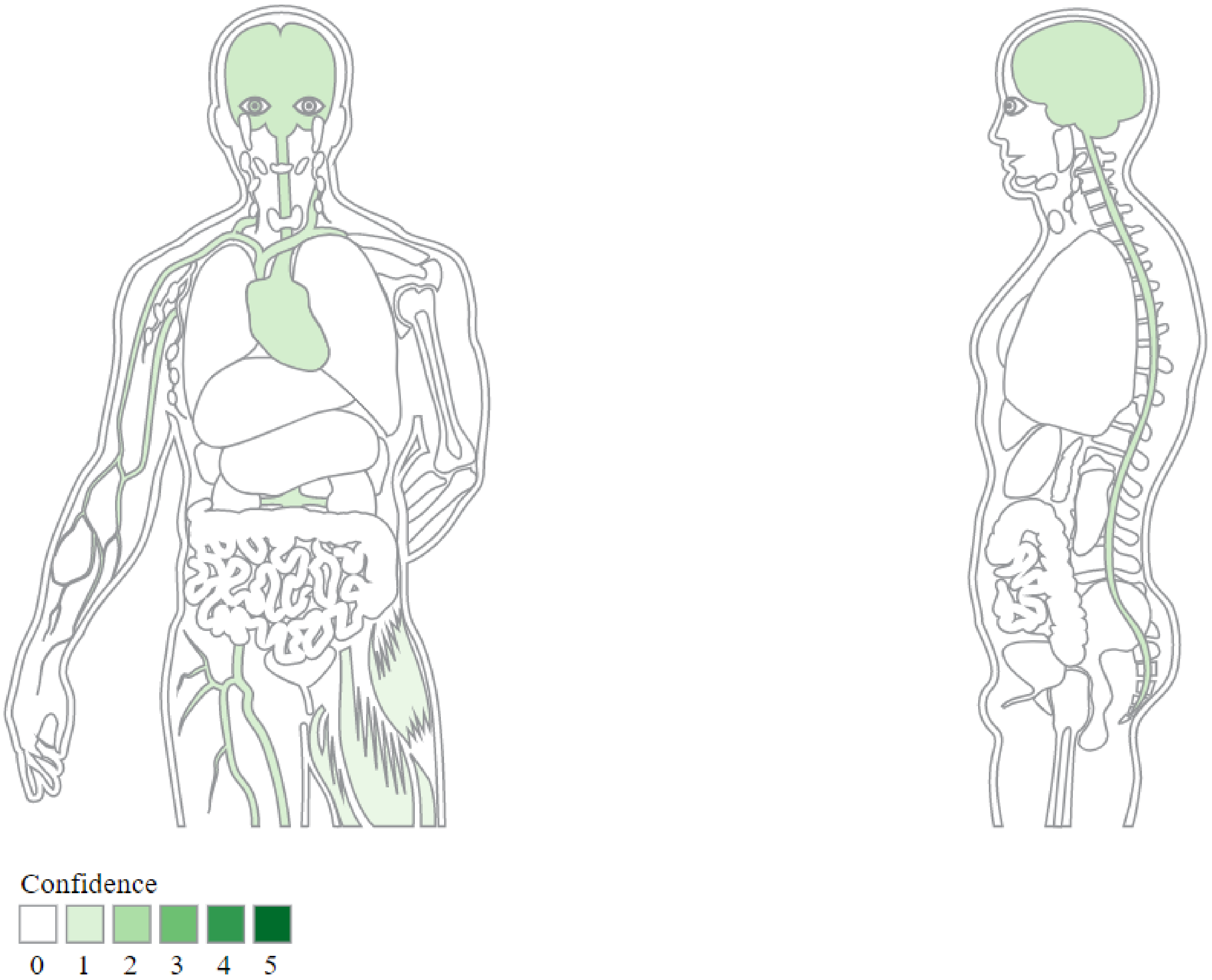

Figure S4. The expression of miR-5096 in different human tissue obtained from TISSUES web resource. The graph represents expression of miR-5096 based on published data.

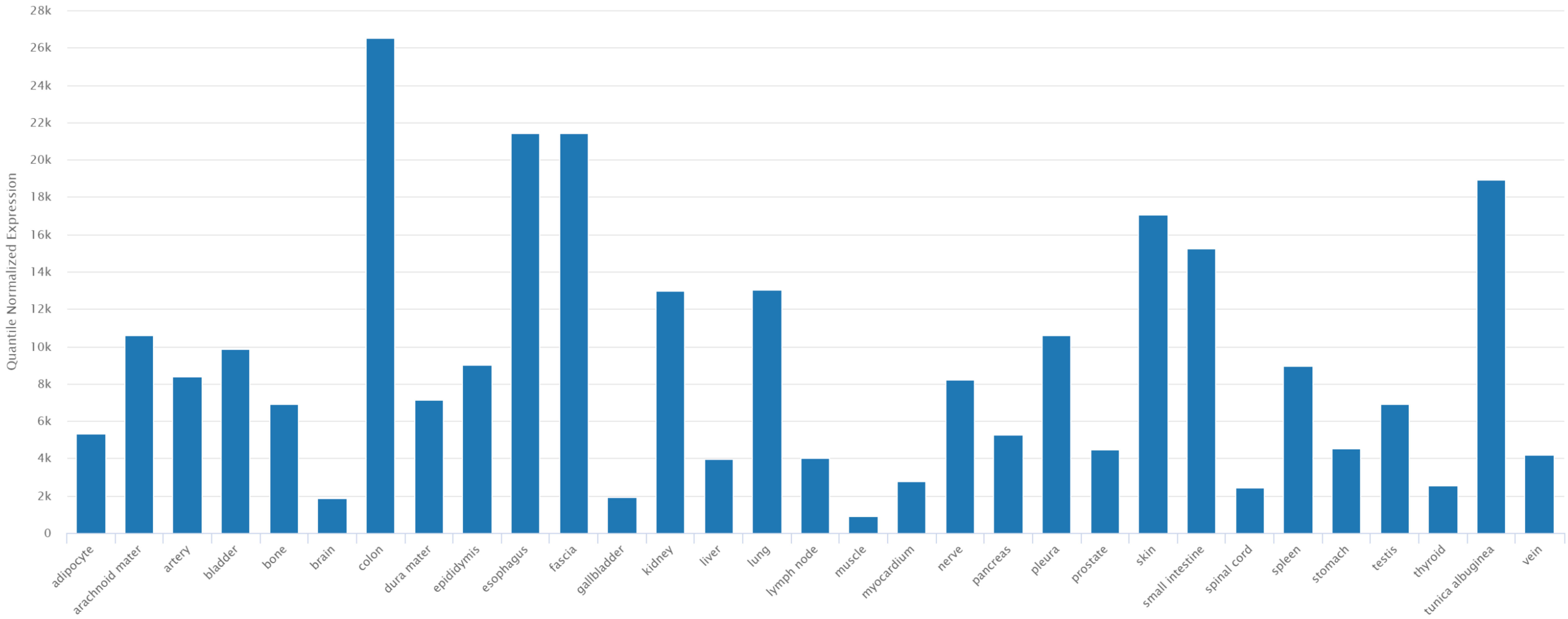

Figure S5. The expression of miR-197-5p in different human tissue obtained from TissueAtlas data base. The graph represents expression level in two different samples.

hsa-miR-197-5p tissues

hsa-miR-197-5p [hsa-miR-197-5p]

Synonyms: hsa-miR-197-5p, MIMAT0022691, miR-197-5p

Linkouts: STRING Pharos

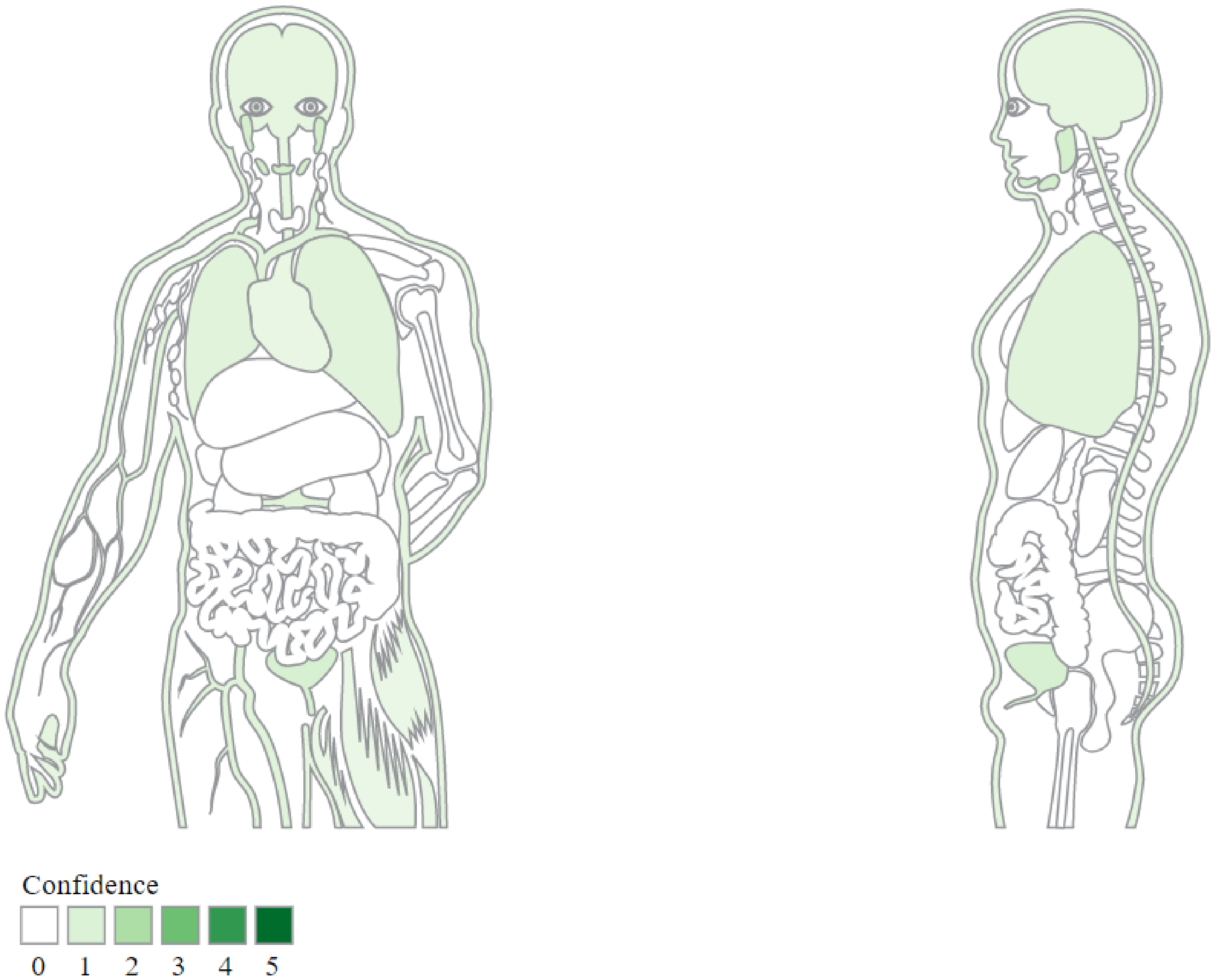

Figure S6. The expression of miR-197-5p in different human tissue obtained from TISSUES web resource. The graph represents expression of miR-197-5p based on published data.

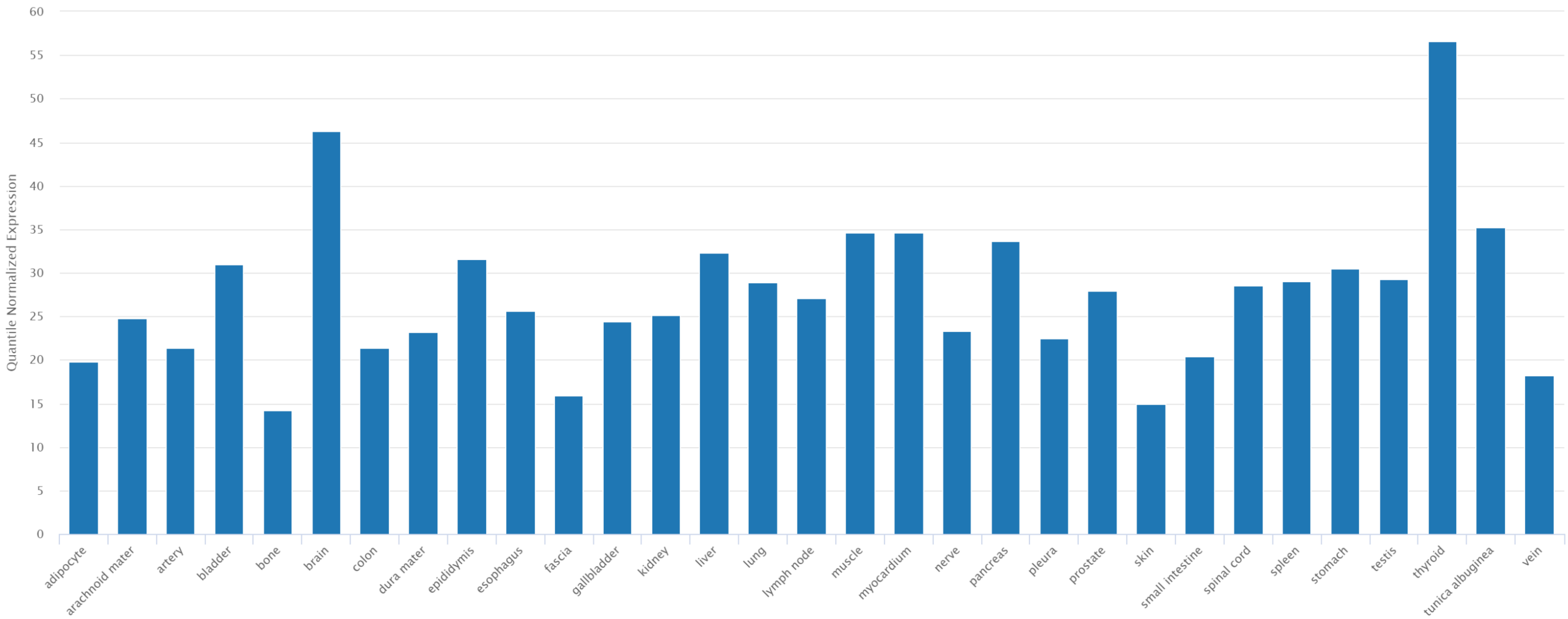

Figure S7. The expression of miR-3935 in different human tissue obtained from TissueAtlas data base. The graph represents expression level in two different samples.

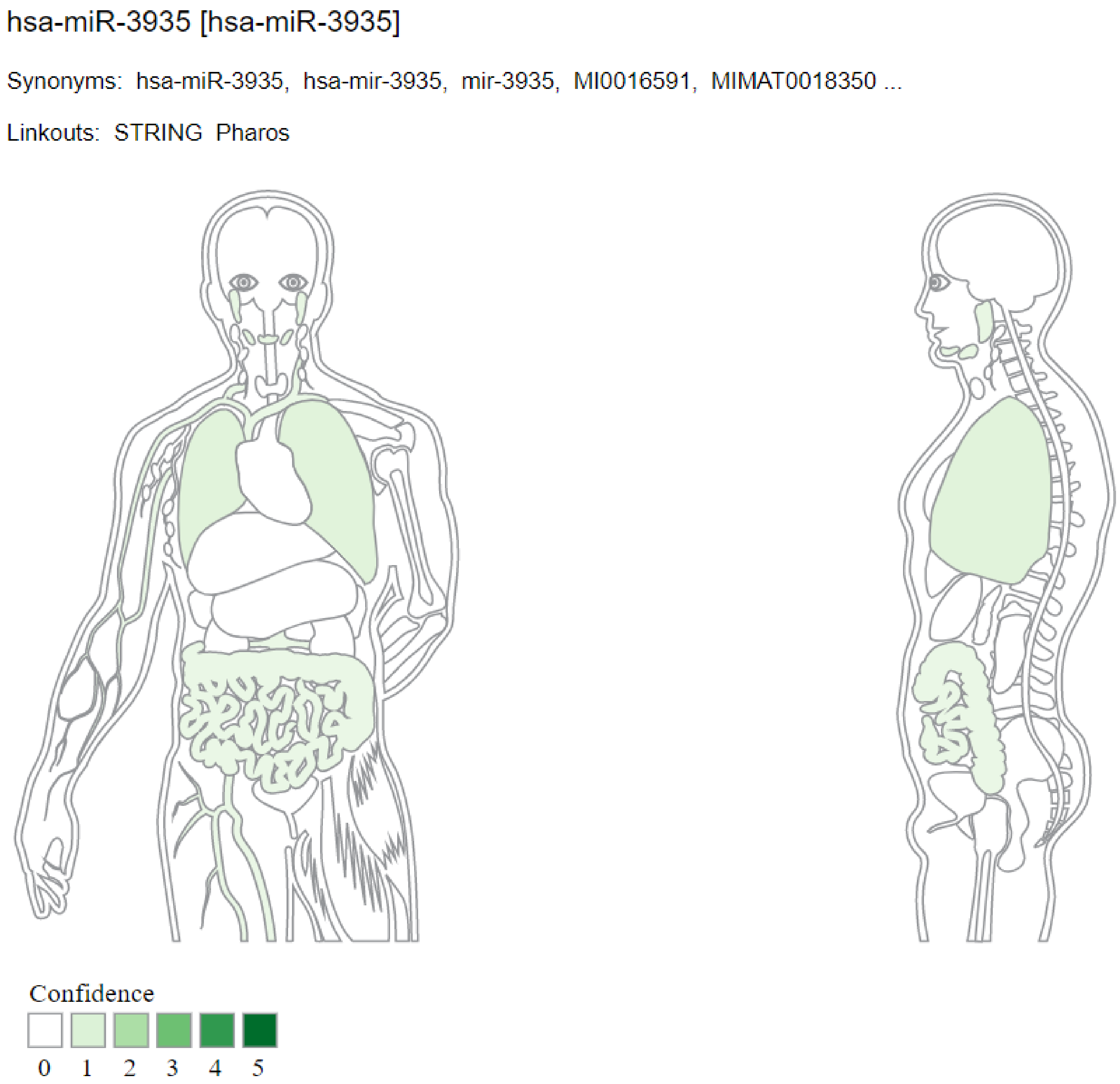

Figure S8. The expression of miR-3935 in different human tissue obtained from TISSUES web resource. The graph represents expression of miR-3935 based on published data.

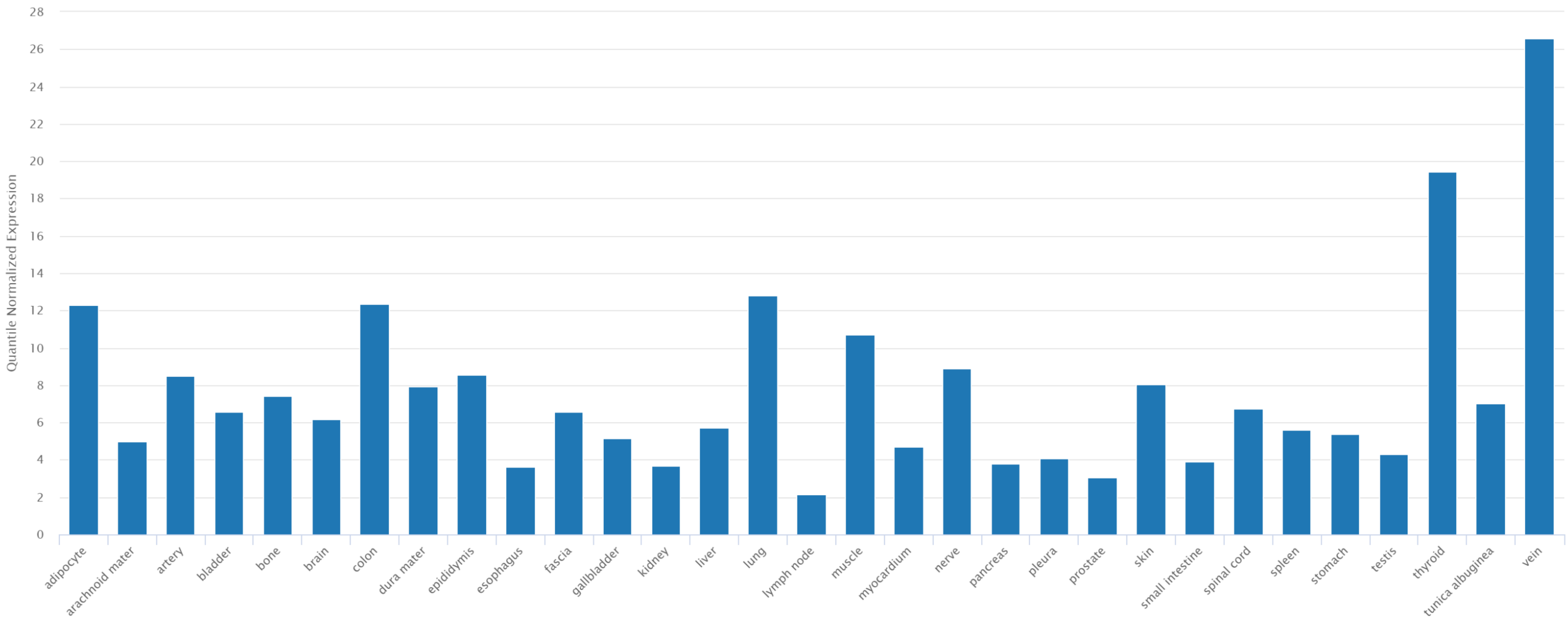

Figure S9. The expression of miR-18b-5p in different human tissue obtained from TissueAtlas data base. The graph represents expression level in two different samples.

hsa-miR-18b-5p tissues

hsa-miR-18b-5p [hsa-miR-18b-5p]

Synonyms: hsa-miR-18b-5p, miR-18b-5p, hsa-miR-18b, MIMAT0001412, miR-18b

Linkouts: STRING Pharos

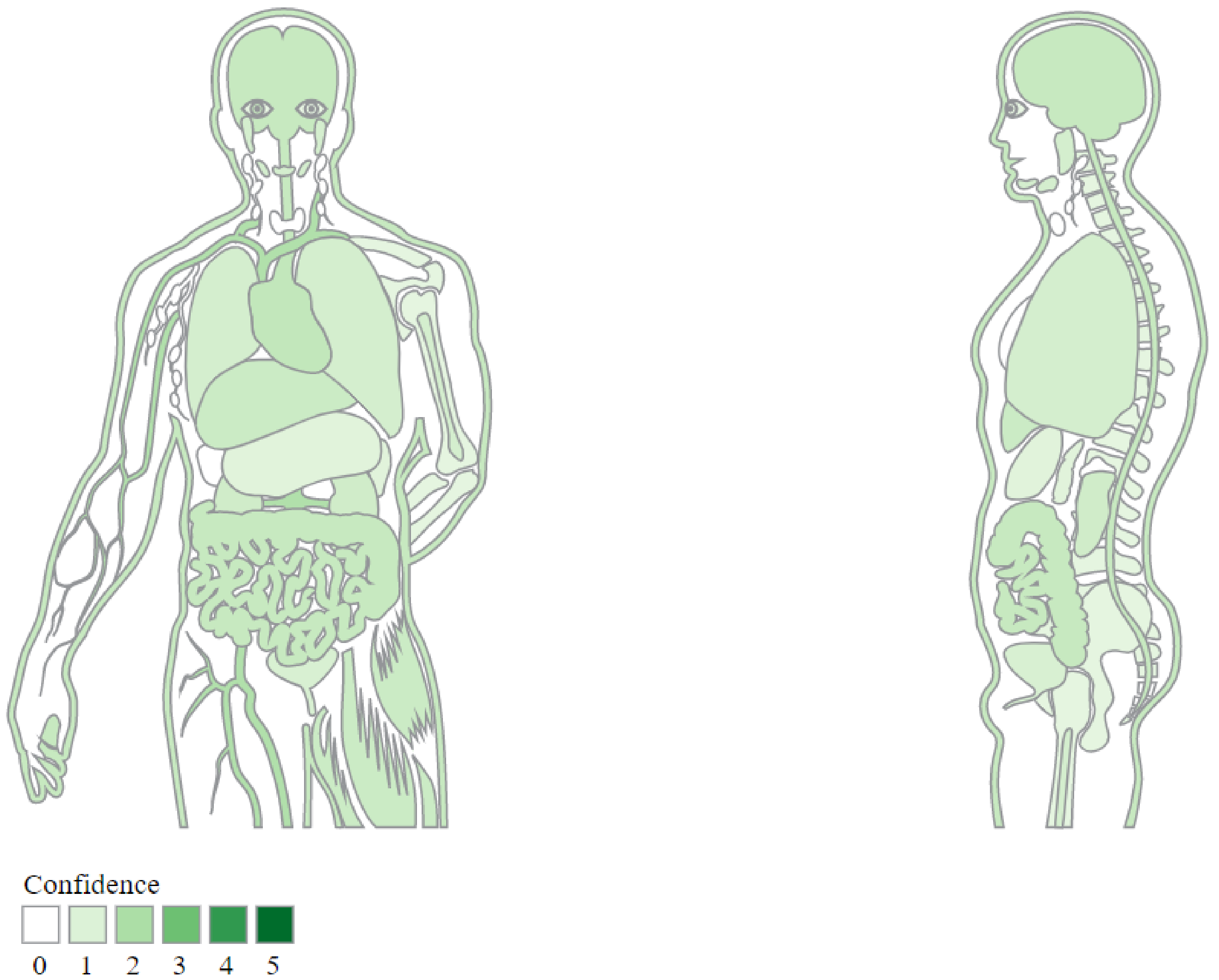

Figure S10. The expression of miR-18b-5p in different human tissue obtained from TISSUES web resource. The graph represents expression of miR-18b-5p based on published data.

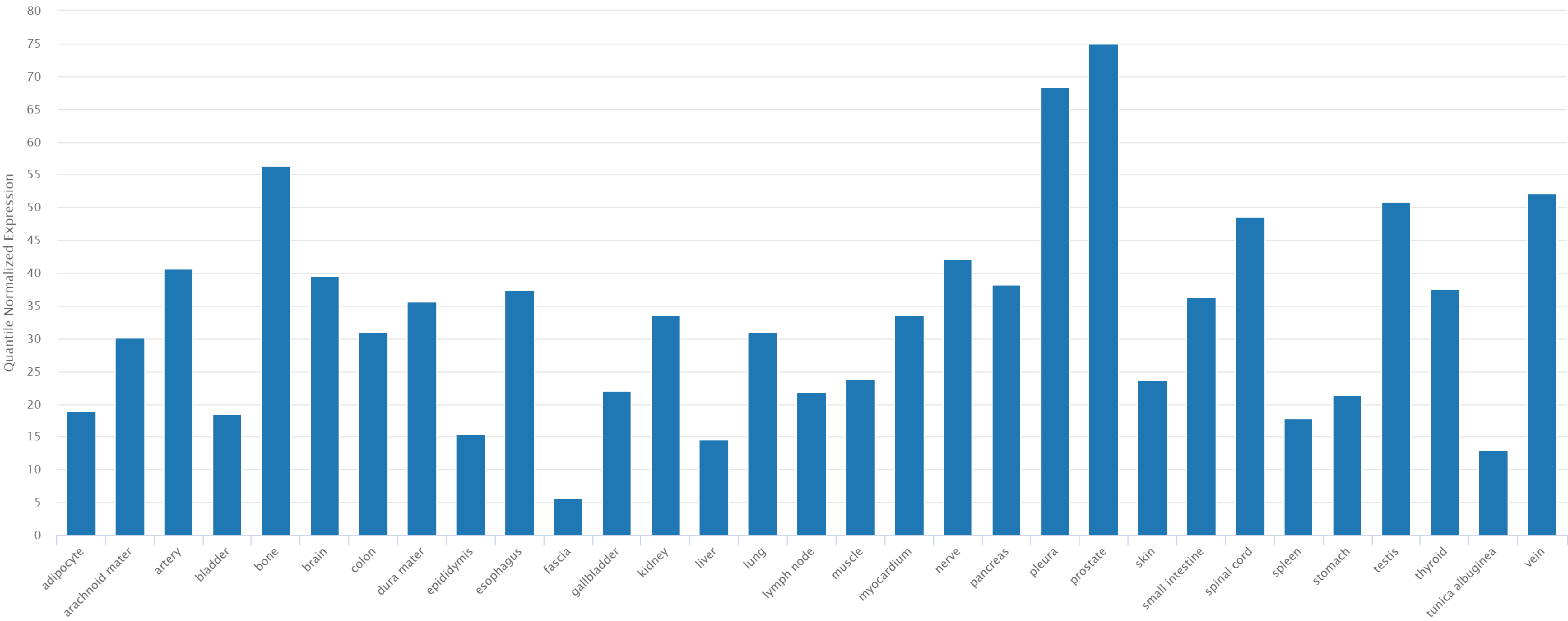

Figure S11. The expression of miR-1273d in different human tissue obtained from TissueAtlas data base. The graph represents expression level in two different samples.

hsa-miR-1273d tissues

hsa-miR-1273d [hsa-miR-1273d]

Synonyms: hsa-miR-1273d, MIMAT0015090, mir-1273d, MI0014254, hsa-mir-1273d ...

Linkouts: STRING Pharos

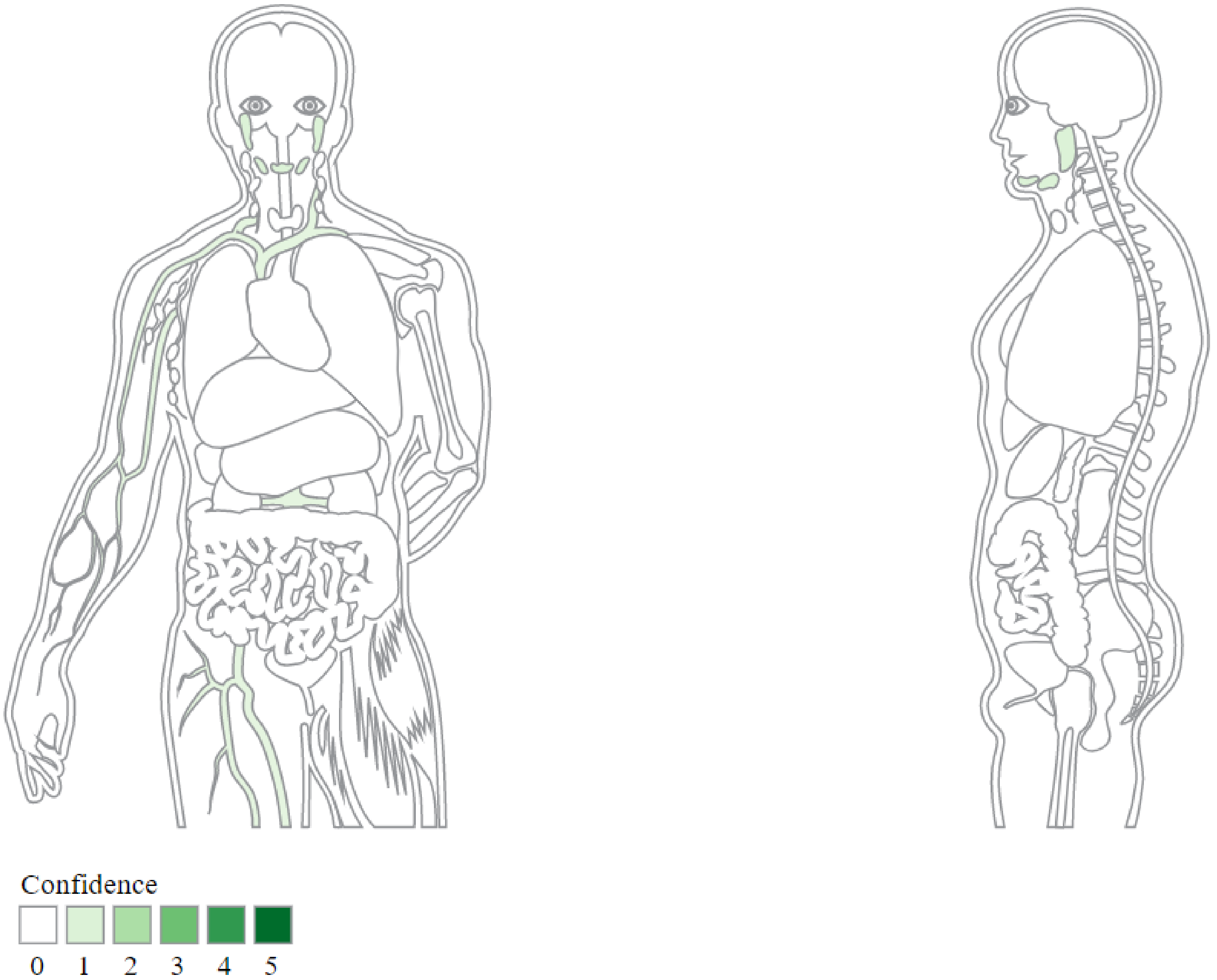

Figure S12. The expression of miR-1273d in different human tissue obtained from TISSUES web resource. The graph represents expression of miR-1273d based on published data.

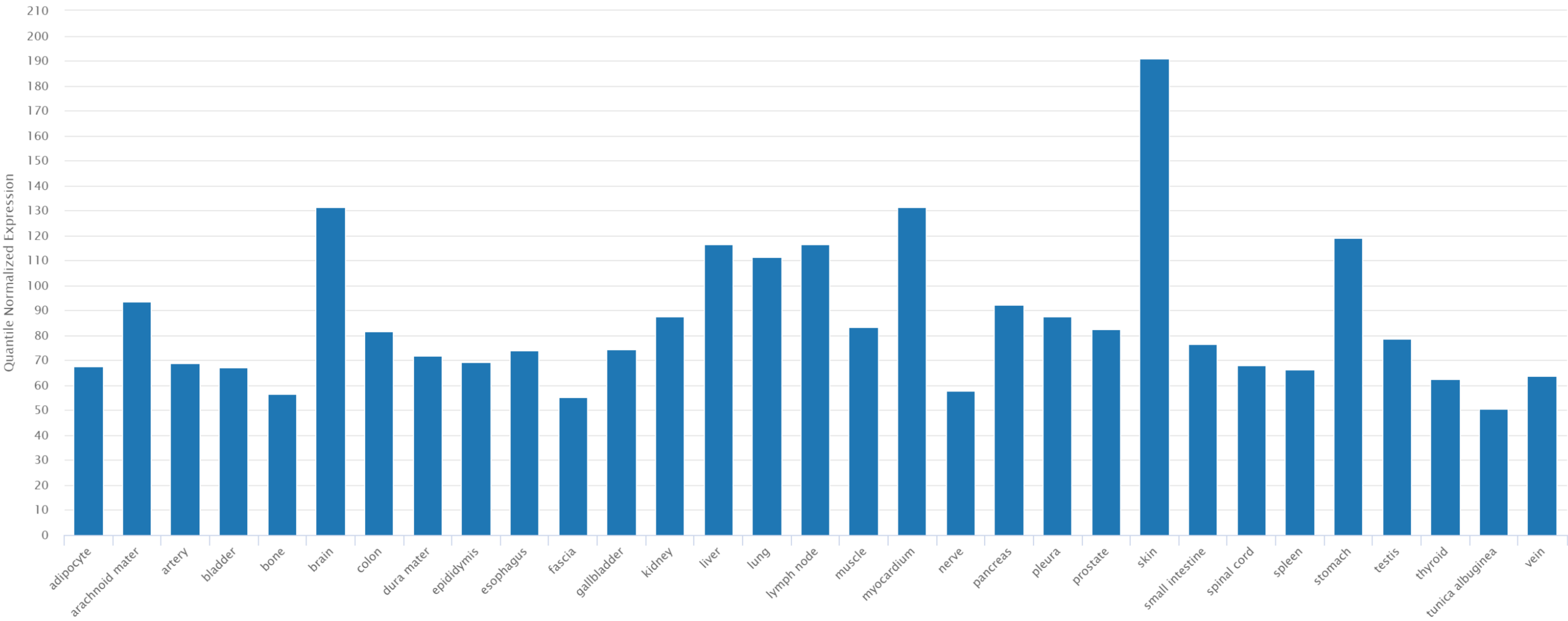

Figure S13. The expression of miR-3154 in different human tissue obtained from TissueAtlas data base. The graph represents expression level in two different samples.

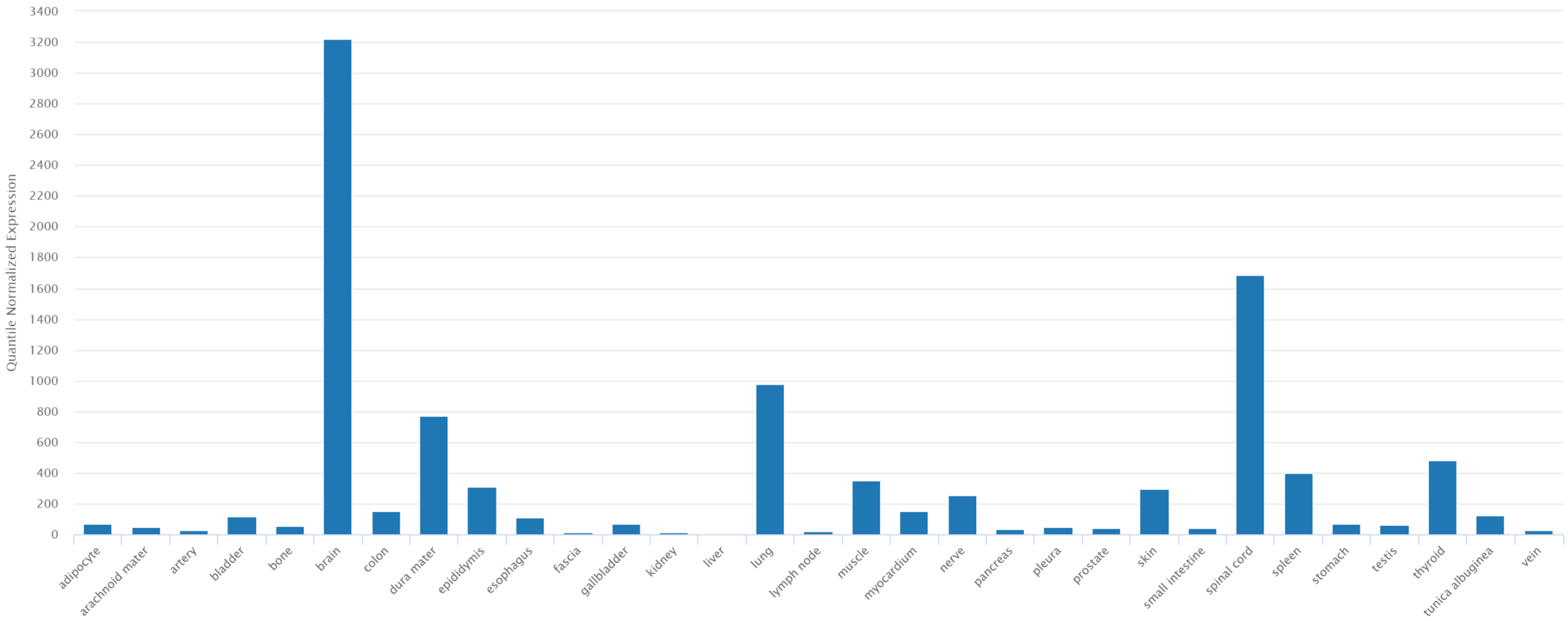

Figure S14. The expression of miR-338-3p in different human tissue obtained from TissueAtlas data base. The graph represents expression level in two different samples.

hsa-miR-338-3p [hsa-miR-338-3p]

Synonyms: hsa-miR-338-3p, miR-338, miR-338-3p, MIMAT0000763, hsa-miR-338

Linkouts: STRING Pharos

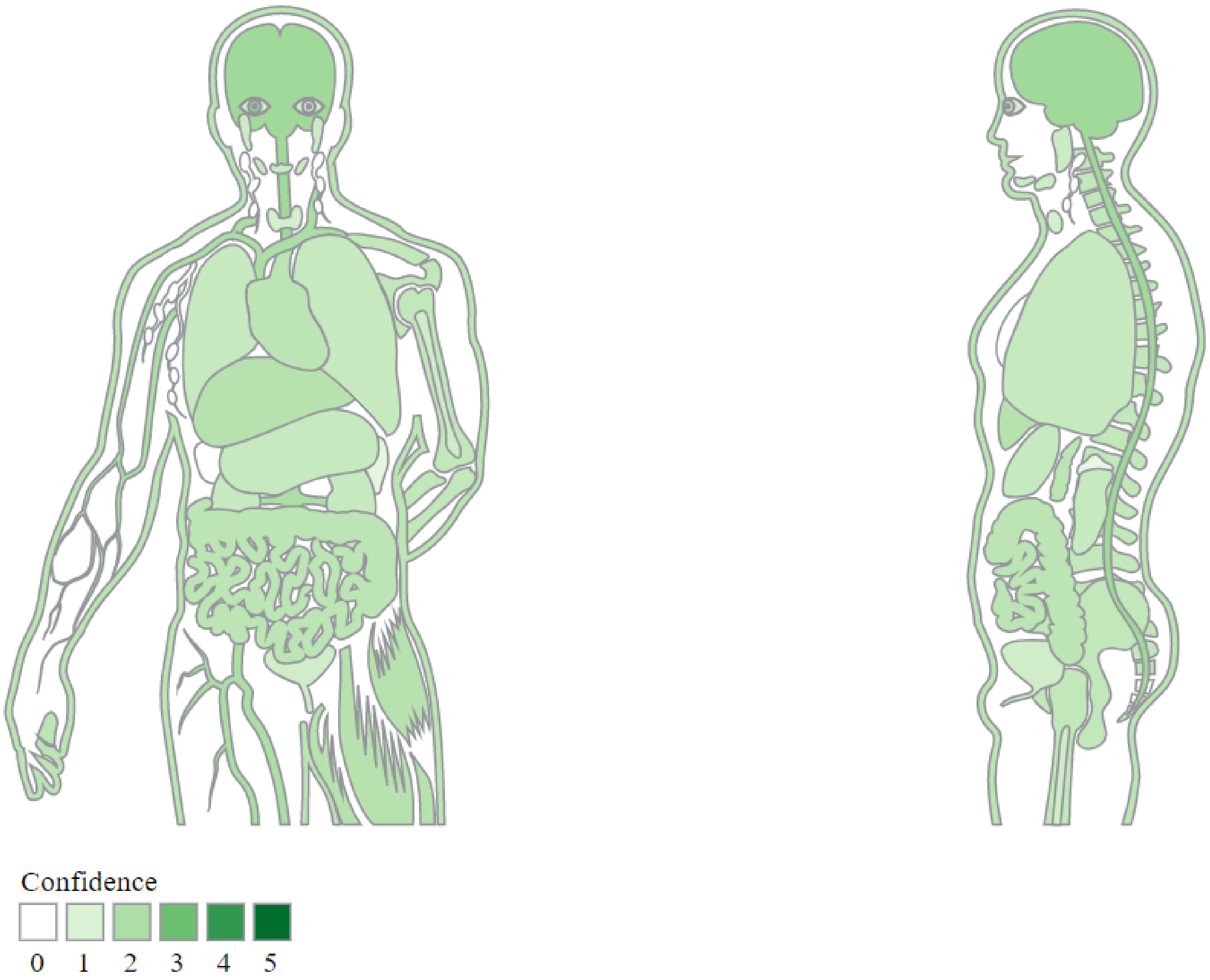

Figure S15. The expression of miR-338-3p in different human tissue obtained from TISSUES web resource. The graph represents expression of miR-338-3p based on published data.

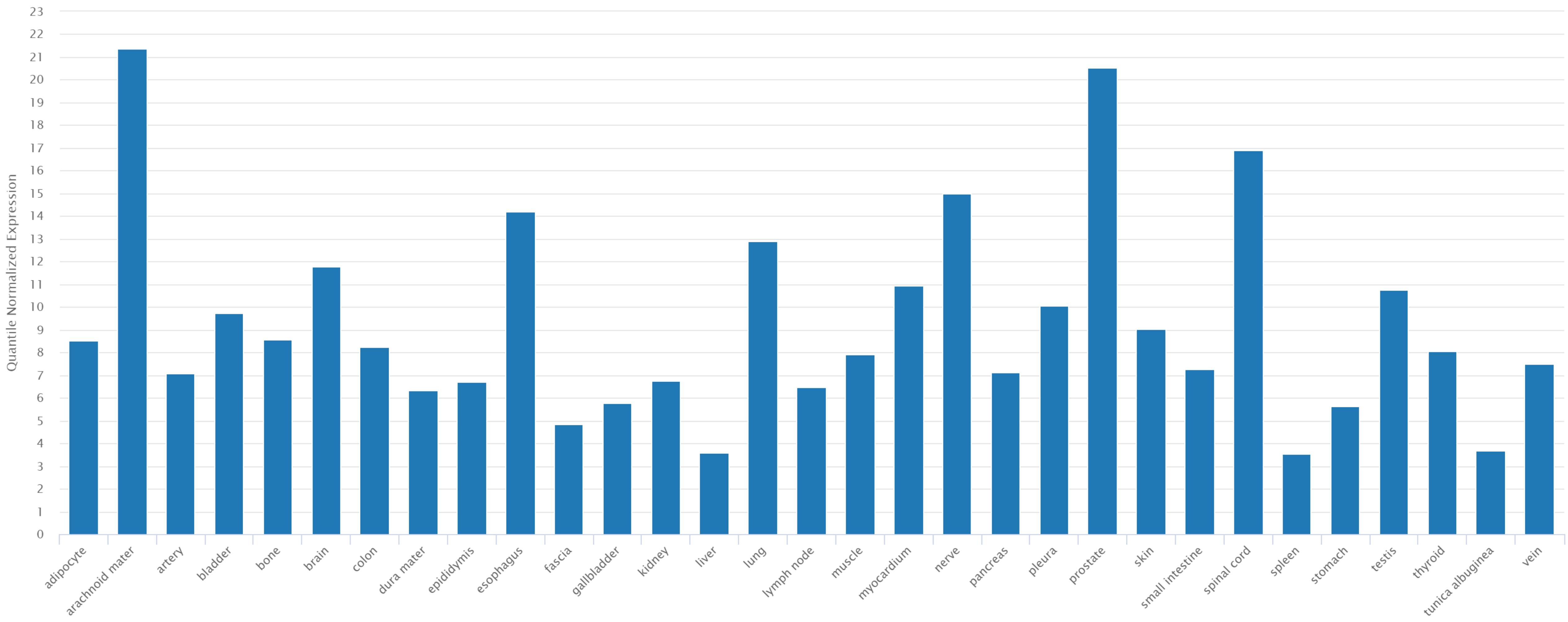

Figure S16. The expression of miR-4436a in different human tissue obtained from TissueAtlas data base. The graph represents expression level in two different samples.

hsa-miR-4436a tissues

hsa-miR-4436a [hsa-miR-4436a]

Synonyms: hsa-miR-4436a, hsa-mir-4436a, MI0016776, mir-4436a, miR-4436a ...

Linkouts: STRING Pharos

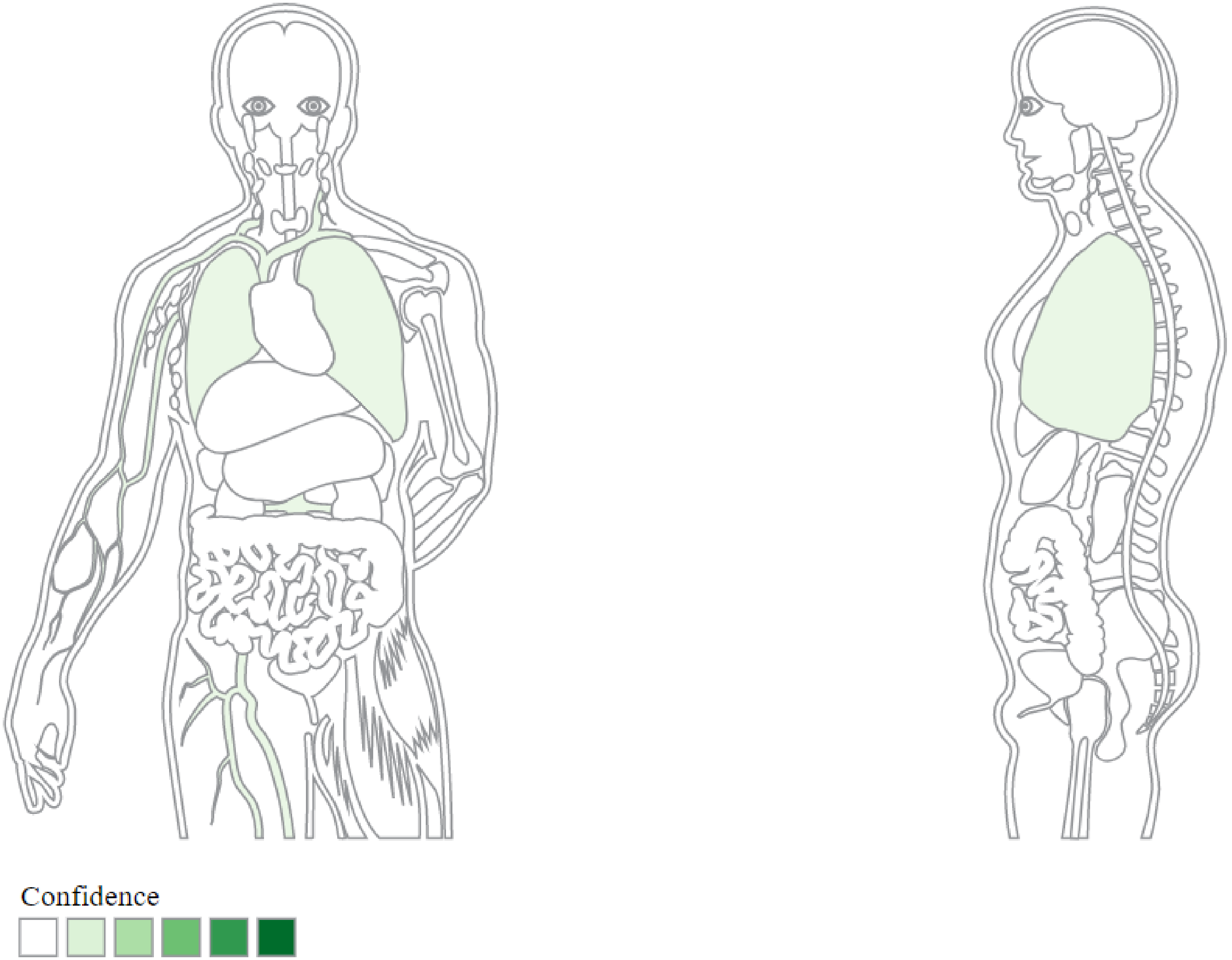

Figure S17. The expression of miR-4436a in different human tissue obtained from TISSUES web resource. The graph represents expression of miR-4436a based on published data.
